# Iron‐associated lipid peroxidation in Alzheimer's disease is increased in lipid rafts with decreased ferroptosis suppressors, tested by chelation in mice

**DOI:** 10.1002/alz.14541

**Published:** 2025-01-29

**Authors:** Max A. Thorwald, Jose A. Godoy‐Lugo, Gilberto Garcia, Justine Silva, Minhoo Kim, Amy Christensen, Wendy J. Mack, Elizabeth Head, Peggy A. O'Day, Bérénice A. Benayoun, Todd E. Morgan, Christian J. Pike, Ryo Higuchi‐Sanabria, Henry Jay Forman, Caleb E. Finch

**Affiliations:** ^1^ Leonard Davis School of Gerontology University of Southern California Los Angeles California USA; ^2^ Department of Pathology and Laboratory Medicine University of California Irvine California USA; ^3^ Department of Pediatrics Keck School of Medicine of the University of Southern California Los Angeles California USA; ^4^ Life and Environmental Sciences Department University of California Merced California USA; ^5^ School of Natural Sciences University of California Merced Merced California USA; ^6^ Dornsife College University of Southern California Los Angeles California USA

**Keywords:** 4‐hydroxy‐nonenal, amyloid, ferritin, deferoxamine, early‐onset familial Alzheimer's disease, ferroptosis suppressor protein 1, glutathione cysteine ligase modulator, glutathione peroxidase 4

## Abstract

**INTRODUCTION:**

Iron‐mediated cell death (ferroptosis) is a proposed mechanism of Alzheimer's disease (AD) pathology. While iron is essential for basic biological functions, its reactivity generates oxidants which contribute to cell damage and death.

**METHODS:**

To further resolve mechanisms of iron‐mediated toxicity in AD, we analyzed *post mortem* human brain and ApoEFAD mice.

**RESULTS:**

AD brains had decreased antioxidant enzymes, including those mediated by glutathione (GSH). Subcellular analyses of AD brains showed greater oxidative damage and lower antioxidant enzymes in lipid rafts, the site of amyloid processing, than in the non‐raft membrane fraction. Apolipoprotein E ε4 carriers had lower lipid raft yield with greater membrane oxidation. The hypothesized role of iron in AD pathology was tested in ApoEFAD mice by iron chelation with deferoxamine, which decreased fibrillar amyloid and lipid peroxidation, together with increased GSH‐mediated antioxidants.

**DISCUSSION:**

These novel molecular pathways highlight iron‐mediated damage to lipid rafts during AD.

**Highlghts:**

- Alzheimer's disease (AD) brains have numerous markers for ferroptosis, including increased lipid peroxidation, reduced antioxidant levels, and increased iron storage.
- Lipid rafts in AD cases have increased oxidative damage and reduced antioxidant enzyme levels and activity which are most severe in apolipoprotein E ε4 carriers.
- Neuronal markers are correlated with lipid peroxidation, antioxidant defense, and iron signaling proteins suggesting that neuronal loss is linked to these events.
- Chelation of iron in the early‐onset familial AD model reduces iron‐mediated lipid peroxidation and fibrillar amyloid.

## 1 BACKGROUND

*Post mortem* Alzheimer's disease (AD) neuropathology is traditionally measured using two protein‐aggregation related biomarkers: accumulation of amyloid beta (Aβ) plaques and neurofibrillary tangles (NFTs). However, < 35% of terminal cognitive decline is attributable to these neuropathological markers and is weakly correlated with cognitive status in two large longitudinal studies,^1^, ^2^ suggesting other pathological events are large contributors. Iron and other transition metals have been implicated in neurodegenerative conditions, including AD,^3^, ^4^ with a recent resurgence due to the discovery of ferroptosis, an iron‐mediated form of cell death.^5^ Ferroptosis serves as an umbrella term for at least one of the following occurrences: loss of antioxidant systems, lethal levels of lipid peroxidation, or blockade of glutathione (GSH) synthesis. Numerous reports assume ferroptosis occurs during AD because these events are observed in AD mouse models^6^, ^7^ and limited numbers of *post mortem* brains.^8^, ^9^, ^10^, ^11^, ^12^ Here, we comprehensively investigate markers of ferroptosis by analysis of antioxidant systems, iron and lipid peroxidation, and GSH synthesis pathways in relation to classic AD neuropathology.

Iron was first linked to AD neuropathology in 1953 by association with senile plaques and neurofibrils.^4^ In the last two decades, associations of iron with AD were strengthened by documentation of increased cerebral microbleeds and decreased blood–brain barrier (BBB) integrity, which increases erythrocytes and subsequently iron in brain parenchyma. Microglial digestion of intravasated blood yields hemosiderin deposits, consisting of aggregated iron and iron‐containing proteins.^13^ Due to their high iron load, microglia have been thought to initiate the ferroptosis cascade.^14^ The accumulation of redox active iron results in oxidative damage to lipids, which is elevated in AD cerebrospinal fluid^15^ and the brain.^16^

Ferroptosis is safeguarded against by antioxidant and iron storage proteins regulated by the transcription factor nuclear factor erythroid 2‐related factor 2 (Nrf2), which is decreased during AD.^17^ Under Nrf2's regulation is glutathione peroxidase 4 (GPx4), which was identified as a key inhibitor of ferroptosis.^18^, ^19^ GPx4 can reduce phospholipid hydroperoxides,^20^ distinguishing it from the other seven GPx family members. GPx4 uniquely interacts with oxidized phospholipids and sterols *within* cell membranes for enzymatic reduction by GSH.^21^ Only one other phospholipid hydroperoxidase exists, peroxiredoxin 6 (Prdx6). Prdx6 is from a different enzyme family implicated in ferroptosis.^22^ For enzymatic reduction, both require GSH, which relies on glutamate‐cysteine ligase catalytic (GCLC) and modifier (GCLM) subunits, critical for GSH homeostasis.^23^ Finally, Nrf2 also regulates iron transport and storage proteins such as ferritin, which helps mitigate lipid peroxidation. While these proteins are central in regulating lipid peroxidation, dozens of other proteins merit consideration.^23^, ^24^

Oxidative damage is one of the most prominent features of AD after classic neuropathology hallmarks. Lipid peroxidation results from oxidation of lipid‐rich regions within cells, such as the lipid rafts (LRs) that reside in the cellular membrane. The LR microdomain is rich in cholesterol, sphingomyelin, and phosphatidylcholine, which allows for tighter packing. This tight packing stabilizes proteins and facilitates signal transduction from the cell surface by receptors.^25^ Also contained in LRs are amyloid precursor protein (APP) and the secretase enzymes that generate Aβ peptides.^26^ A loss of the LR alongside increased Aβ peptide generation has been observed in *post mortem* AD brains.^27^ Whether increased oxidation to the LR occurs during AD and what antioxidant enzyme systems protect the LR remain unexplored.

While ferroptosis is implicated in AD neuropathology,^28^, ^29^, ^30^ there are limited data on the levels of ferroptotic proteins^8^, ^31^, ^32^ or tissue iron^8^, ^9^ in the human brain. Experimental knockdown of GPx4 in mice caused neuronal death, supporting the concept of ferroptosis,^33^ and is frequently cited in reviews as evidence for ferroptosis in human AD. Furthermore, neuronal cultures exposed to Aβ42 exhibit reduced GPx4 levels^34^ strengthening the connection between ferroptosis and AD. While GPx4 has an essential role in neuronal survival, AD brains have not been comprehensively examined for GPx4 or other antioxidant processes relevant to ferroptosis. Moreover, xenografting human AD neurons into mice caused necrotic cell death, but not apoptosis or ferroptosis,^35^ while a murine model of acute kidney disease suggested the intertwinement of necroptosis and ferroptosis,^36^ making the role of ferroptosis during AD less clear. The largest study to date considering ferroptosis for human AD brains showed that lipid peroxidation product 4‐hydroxy‐nonenal (HNE) was increased and had differing changes in few ferroptosis‐related proteins.^8^ This and other reports are limited by the small sample size, without accounting for sex or apolipoprotein E (*APOE*) allele, which are both known to modulate AD risk.^37^, ^38^ Finally, a potential confound of prior studies is residual blood from unperfused brains. Blood contains several‐fold higher levels of heme‐iron, iron‐bound proteins, and GSH‐using enzymes than the brain tissue itself.

To accurately examine ferroptotic changes in the brain occurring during AD, we developed a washing protocol for *post mortem* brains that minimizes residual blood. We subsequently measured > 20 proteins related to iron metabolism, antioxidant signaling, and amyloid pathology. We hypothesized that changes consistent with ferroptosis would be identified in AD brains: specifically, that antioxidant systems that protect against lipid peroxidation would be decreased, while the LR, which processes APP, would display extensive oxidative damage. To assess this, a large set of brains from cognitively normal and AD individuals were examined, with equal sex and known *APOE* alleles. We further examined differences between case‐matched prefrontal cortex and the “AD resistant” cerebellum.^39^ The LR was analyzed for antioxidants relevant to ferroptosis, as a potential link between iron‐mediated lipid peroxidation and Aβ generation. Finally, we validated the role of iron in Aβ pathology in an AD mouse model by chelation with deferoxamine (DFO) used in clinical trials for AD.^40^, ^41^ These findings document the loss of antioxidant enzymes in LRs and associated increases in lipid peroxidation during AD.

## 2 METHODS

### 2.1 Human samples

*Post mortem* prefrontal cortex and cerebellum with Braak scores were provided by three Alzheimer's Disease Research Centers (ADRCs): University of Southern California (USC; Dr. Carol Miller), University of California Irvine (UCI MIND), and University of Washington (BRaIN). Samples were genotyped for *APOE* alleles by polymerase chain reaction for single nucleotide polymorphism variants rs429358 and rs7412. All human subjects provided informed consent. Human tissue use was approved through institutional review board protocol #UP‐20‐00014‐EXEMPT. Details for individual brains are in Table S1 in supporting information.

### 2.2 Mice

C57BL/6 mice from Jackson laboratories or ApoEFAD mice from Mary Jo LaDu (University of Illinois, Chicago, USA) were housed by the University of Southern California's Department of Animal Resources. Mice had ad libitum access to Purina Lab Chow (LabDiet) and water. Mice were housed in groups of five at 22°C/30% humidity, with standard nesting material and light cycles of 6:00 am to 6:00 pm. Studies were approved by the USC Institutional Animal Care and Use Committee (IACUC# 20417). Female ApoEFAD mice were given DFO treatment either in rodent chow (10 mg/kg/day) for 2 weeks or by intraperitoneal injection twice daily for 1 week (10 mg/kg/day). Six‐month‐old mice were anesthetized with isoflurane and euthanized by cardiac puncture. C57BL/6 mouse cortex was collected as non‐perfused, with transcardial phosphate‐buffered saline (PBS) perfusion, or PBS perfusion + 10U/mL heparin (Sigma‐Aldrich). Tissues were stored at –80°C.

**RESEARCH IN CONTEXT**

1. **Systematic review**: Iron is implicated in Alzheimer's disease (AD) pathology through its role in oxidation of lipids and association with amyloid plaques. The hypothesis of ferroptosis has been proposed for AD, but little primary evidence exists in the literature for human tissues.
2. **Interpretation**: Alterations in iron metabolism and decreases in antioxidant defense confirm a loss of mechanisms that protect lipid rafts, the site of amyloid processing during AD. Ferroptotic events may exacerbate amyloid pathology, which is attenuated in AD mice.
3. **Future directions**: Iron chelation therapies in combination with amyloid monoclonals may be considered. Redox active iron is liberated from amyloid fibrils, which may cause oxidative bursts.

### 2.3 Tissue washing

Human and mouse brain tissue was thawed on ice and minced using a ceramic scalpel for iron and heme measurements or by surgical scissors. PBS (10 volumes) was placed in each tube and the samples were gently washed by two cycles of vortexing and collected by gentle centrifugation at 800 x g/30 seconds/4°C. PBS was retained on a small subset of samples and further spun at 4000 x g/10 minutes/ 4°C. The pellet was sonicated for 10 seconds at 50% power in PBS. Resuspended pellet and lysate were assayed for heme (Table 1).

**TABLE 1:** Heme and total iron in the prefrontal cortex were assayed for effects of washing to minimize residual intravascular blood: (A) mouse (C57BL/6J, 6 month; *n* = 5–6); (B) human (AD; *n* = 24) and controls (65+ years; *n* = 24) prefrontal cortex (Brodmann regions 8–10); (C) iron^9^, ^42^, ^43^ and ferritin^43^, ^44^, ^45^, ^46^ in brain, blood, serum, and CSF.

A. Mouse, wildtype				
	Non‐perfused	Perfused PBS	Perfused PBS + heparin	Non‐perfused + washed
Heme/mg wet brain	3.1 ± 0.2	2.1 ± 0.2* (‐32%)	2.0 ± 2.3** (‐35%)	1.3 ± 0.2*** (‐68%)

B. Human		
Source	Unwashed	Washed
Total iron, ug/g wet brain	45 + 4.9	26 + 1.4 ****, ‐45%
Ayton et al. 2021^9^	≈ 55	N/A
Ashraf et al. 2020^8^	≈ 290	N/A
Heme/mg	7.1 + 0.4	3.6 + 0.2, ****, ‐50%
a‐Hemoglobin, µg/mg	1,644 + 131	815 + 61, ****, ‐50%
Ferritin light chain (RFUs)	235 + 17	100 + 9, **** ‐60%

C. Sources of iron and ferritin		
Compartment	Total iron	Ferritin
Brain, unwashed	0050 µg/g wet brain	0.135 µg/g
Blood	3500 ug/mL	3.8‐14 µg/mL
Serum	001.3 ± 0.14 µg/mL	0.011‐3.4 µg/mL
CSF	0.061 ± 0.006 µg/mL	0.012 µg/mL

*Notes*: The ferritin complex in erythrocytes and tissue includes FTL and FTH1. Isotonic PBS (pH 7.4) was used for aortic perfusion for mice and washing of minced mouse and human brains. Thirty milligrams of prefrontal cortex was minced into 2 mm cubes and “washed” by gentle vortexing in PBS. Heme and α‐hemoglobin per mg tissue were assayed spectrophotometrically relative to unwashed brains (Figure S3 in supporting information). Total iron levels from Ashraf^8^ were estimated as Fe/g protein, assuming 1 g protein/12 g wet weight brain. Brain iron and ferritin by compartment for humans are > 95% below blood levels, assuming 1 mL volume is equivalent to 1 g wet brain.Significance by one‐way analysis of variance (mouse) with Tukey post hoc, or two‐tailed *t* test (human): *P < 0.05, **P < 0.01, ***P < 0.001, ****P < 0.0001.

Abbreviations: AD, Alzheimer's disease; CSF, cerebrospinal fluid; FTH1, ferritin heavy chain 1; FTL, ferritin light chain; PBS, phosphate buffered saline; RFU, relative fluorescent unit.

### 2.4 Amyloid measurements

Human tissue was processed for amyloid measurements as previously described.^27^ Briefly, tissue was homogenized in RIPA without sodium dodecyl sulfate and spun for 10,000 x g for 1 hour at 4°C. The supernatant was collected and analyzed for soluble Aβ fibrils using M78 (conformation specific antibody for Aβ fibrils,^48^ a gift from Dr. Charles Glabe, UCI). The residual pellet was resuspended in 70% formic acid and sonicated. The resuspension was nutated at room temperature for 2 hours before neutralization with 20 volumes of 1 M Tris and concentrated with a vacuum concentrator (Labconco). Amyloid levels were assayed by dot blot and visualized as described below for Western blots.

### 2.5 LR and non‐raft membrane

The LR fraction was isolated from 40 mg tissue from frozen prefrontal cortex (Brodmann area 8, 9, or 10) and cerebellum using a column‐based isolation kit (Invent Biotechnologies). These findings were verified by sucrose gradient ultracentrifugation.^49^ The LR and the remaining detergent soluble non‐raft membrane (NRM) fractions were resuspended in PBS+1% triton x‐100 and sonicated for 10 seconds at 50% power. Protein was assayed by the copper‐based 660 nm assay (ThermoFisher Scientific). LR enrichment and purity were determined by cytosolic glyceraldehyde‐3‐phosphoate dehydrogenase (GAPDH), several LR markers including flotillin1, and nuclear marker H3.^27^

### 2.6 Nuclear isolation

Twenty mg of frozen brain was homogenized in 125 µL of sucrose buffer for nuclear and cytosolic fractions. A previously published protocol^50^ was modified for brain tissue by adding 1% NP‐40 to the first wash step, resuspending the pellet, and incubating on ice for 30 minutes. Nuclear and cytosolic extractions were cross‐probed for purity against both H3 and GAPDH by western blot^51^ and showed the expected enrichment (Figure S1 in supporting information).

### 2.7 Western blot

Proteins from LRs (5 µg), whole cell lysate (20 µg), or nuclear fractions (20 µg) were heated at 75°C under denaturing conditions and resolved on 4% to 20% gradient gels. Proteins were electroblotted using a Criterion blotter (Bio‐Rad Laboratories) and transferred onto 0.45 µm polyvinyl difluoride membranes. Membranes were stained using Revert 700 fluorescent protein stain and imaged before blocking with LI‐COR Intercept blocking buffer (LI‐COR Biosciences), followed by primary antibodies. Membranes were incubated with IRDye 800CW and/or 700CW secondary antibodies and visualized by Odyssey (LI‐COR Biosciences).

### 2.8 Western and dot blot analysis

Densitometry values were calculated from fluorescent signals on western blot and dot blot images by ImageJ. Raw densitometry was normalized by total protein per lane detected by Revert stain and/or loading control protein. Histograms are presented as percent change from control labeled as relative fluorescent units (RFUs).

### 2.9 Biochemical assays

LRs were assayed for total cholesterol (Cell Biolabs). Tissue hemoglobin or heme were quantified by Quantichrom assay (BioAssay Systems). α‐Hemoglobin was quantified by enzyme‐linked immunosorbent assay (ThermoFisher Scientific). Total glutathione peroxidase activities were measured by enzymatic assay (Cayman Chemical). GPx4 activity was assayed by generating oxidized phosphatidylcholine using soybean lipoxygenase.^52^ Purified phosphatidylcholine hydroperoxide was used in place of cumene hydroperoxide in Cayman's total GPx activity assay at a concentration of 30 µM per well.

### 2.10 Inductively coupled plasma mass spectrometry

Fifty milligrams of brain tissue was cut with a ceramic scalpel and placed in metal‐free certified test tubes. Tissues were washed twice with PBS and homogenized in PBS by polyethylene pestle (PES‐15‐B‐SI, Axygen) with purified water (18.2 MΩ; Millipore) treated with Chelex 100 (sigma). Homogenates were desiccated by vacuum centrifuge at 95°C for 90 minutes and solubilized in trace metal free 70% HNO_3_ overnight. Thirty percent H_2_O_2_ was then added followed by desiccation. After resuspension in 2% HNO_3_, samples were analyzed by Agilent 7500ce inductively coupled plasma mass spectrometry (ICP‐MS) in hydrogen mode with a practical detection limit of 10 ppb and a relative standard deviation (RSD) of replicate measures between 0.2% and 5%. Total iron concentration was normalized to wet weight tissue.

### 2.11 RNA‐sequencing

Twenty milligrams of brain tissue was homogenized in TRIzol reagent using a BeadBug Benchtop Homogenizer. Brain tissue was suspended in 1 mL of TRIzol and homogenized for six rounds of 10 second homogenization and 60 second holds between each round. Three hundred microliters of chloroform was added to the sample and aqueous separation of RNA was performed using centrifugation in a heavy gel phase‐lock tube (VWR, 10847‐802). The aqueous phase was applied to a standard column‐based RNA purification kit (Quantabio, Extracta Plus, Cat# 95214‐050) following the manufacturer's protocol. Library preparation and RNA‐sequencing (RNA‐seq) were performed by Novogene. Reads from samples were processed using trim_galore (0.6.5‐1) and mapped to the GRCh38.p14 genome with STAR‐2.7.10a. Mapped reads were counted to genes using featureCounts (Subread‐2.0.3) and the GRCh38.108 GTF file. Unwanted noise and artifacts were removed using svaseq and removeBatchEffects from the sva package‐3.42.0 and differential expression analysis was performed with DESeq2‐1.34.0 using R‐4.3.2. Raw RNA‐seq data are available through Annotare: E‐MTAB‐14167.

### 2.12 Statistics

GraphPad Prism 9 software (GraphPad) was used for graphing and statistical analysis. Several measures were adjusted by age, sex, PMI, ADRC source, and presence of vascular comorbidities by multiple linear regression, which yielded no significant interactions. For these reasons, data were analyzed by two‐tailed *t* test or one‐way analysis of variance with Tukey honestly significant difference (*P* < 0.05) with a confidence interval set at 95%. Data that were not normally distributed were analyzed by Mann–Whitney *U* or Kruskal–Wallis with Dunn test. All correlation matrixes used Spearman correlation for non‐linear data.

## 3 RESULTS

### 3.1 Minimization of residual blood in *post mortem* brain

Studies of iron in *post mortem* tissues contain intravascular blood, rich in iron proteins. In rodent studies, blood can be removed by cardiac perfusion with heparinized saline, which we show decreased heme iron in the cerebral cortex by 35% (Table 1A). Because perfusion is not feasible for thawed human cerebral cortex, we developed a washing procedure to minimize adherent and entrapped vascular blood. Thawed brain was minced into 2 mm cubes, followed by gentle washing with isotonic buffer (PBS). “Washed” tissues had 65% less heme than the non‐perfused, displaying less heme even compared to perfused samples that were not washed (Table 1A), suggesting most heme is from residual blood on tissue rather than in the vasculature.

For human cortex (Table 1B), our washing protocol decreased levels of heme by 50%. As expected, washing reduced total iron levels significantly in the cortex (Table 1B, Figure S2A in supporting information). Importantly, total iron levels in unwashed samples were similar to those found in previous reports which observe increased brain iron with AD, suggesting that residual blood skews iron levels.^8^, ^9^, ^53^ Total aluminum, copper, and zinc, which generally have low levels in the blood, were not altered by washing (Figure S2B‐D), supporting that our washing protocol was specific to removing blood.

We further validated our washing protocol by centrifugation of the residual PBS wash from tissues. The remaining supernate contained heme levels equivalent to the difference between washed and unwashed tissue (Figure S3A in supporting information). Serial washes were performed, which showed diminishing decreases in heme concentrations after the second wash (Figure S3B). For these reasons, tissues were washed twice for the remaining measurements. α‐hemoglobin and ferritin light chain, two markers for erythrocytes, showed decreased levels > 50% in our washed samples (Figure S3C‐F). While our data suggest that some residual blood still exists in samples after washing, our protocols eliminated blood contaminants by at least half. Therefore, for the entirety of this study, all analyses were performed on washed samples to minimize this confounding variable.

### 3.2 Lipid peroxidation increased in AD

**FIGURE 1:**
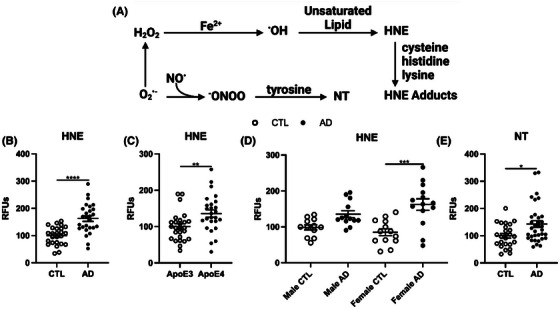
Oxidative damage (HNE, NT) to prefrontal cortex from age‐matched AD (*n* = 27) and cognitively normal controls (CTL; *n* = 25). (A) Schematic representing oxidative damage to lipids and proteins. Hydroxyl radicals (^.^OH) are produced during oxidation of Fe^2+^ or Cu^2+^ (“Fenton chemistry”) and may oxidize adjacent proteins, lipids, or nucleic acids. HNE can alter proteins via Michael addition products through its double‐bonded carbons, or by forming Schiff bases through its aldehyde moiety. The bottom line shows the iron‐independent oxidative pathway to form peroxynitrite ^−^ONOO from O_2_^•–^ and ^.^NO, which can then oxidize tyrosine to NT. Aggregated human Aβ42 stimulated the formation of peroxynitrite which is neurotoxic in vitro.^55^ Non‐heme iron also mediates lipoxygenase activity.^56^ Dot blots are presented as percent change from control for RFUs for HNE presented as (B) cognitively normal versus AD, (C) *APOE* allele, (D) sex, and (E) NT. Significance, two‐tailed *t* test (B,C, E), one‐way analysis of variance with Tukey post hoc test (D): *P < 0.05, **P < 0.01, ***p < 0.001, ****p < 0.0001. AD, Alzheimer's disease; *APOE*, apolipoprotein E; HNE, HNE, 4‐hydroxy‐nonenal; NT, 3‐nitrotyrosine; RFU, relative fluorescent units.

First, we measured levels of lipid peroxidation, a principal component of ferroptosis described as the oxidation of lipids by free radicals (Figure 1A), previously observed in other AD tissues.^54^ Oxidative damage was assessed in the prefrontal cortex by two protein adducts, HNE and 3‐nitrotyrosine (NT). AD samples exhibited a significant increase in both HNE and NT compared to controls (Figure 1B,C), suggesting increased lipid peroxidation is correlated with AD pathology. To determine whether these changes were associated with *APOE* status or sex, we next measured HNE and NT in *APOE* ε3 versus *APOE* ε4 carriers and found that lipid peroxidation was higher in *APOE* ε4 carriers, consistent with previous reports that *APOE* ε4 carriers are at higher risk for pathology (Figure 1D). When separated by sex, females displayed an increase in HNE, but males showed a trending, but not significant, increase in HNE, also consistent with previous reports that females display worse AD pathology than males^38^ (Figure 1E).

**EXTENDED DATA FIGURE 1 alz14541-fig-0009:**
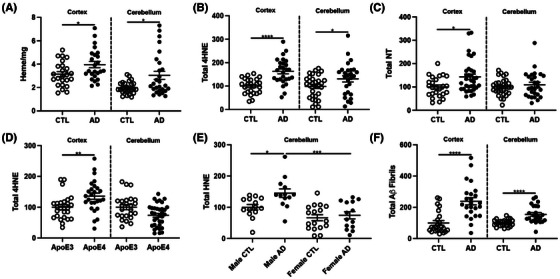
Oxidative damage (HNE, NT) in human prefrontal cortex and cerebellar proteins in age matched AD and cognitively normal control (CTL). Prefrontal cortex from Brodmann area 8, 9, or 10 and cerebellum were washed for measurements of (A) heme, (B) dot blot for 4HNE, (C) dot blot for NT, (D) HNE, (E) HNE, (F) total amyloid fibrils. Percent change from control as RFUs. Significance, two‐tailed t‐test (A‐D, F), one‐way analysis of variance with Tukey post hoc test (E): ^*^ *p* < 0.05, ^**^ *p* < 0.01, ^***^ *p* < 0.001, ^****^ *p* < 0.0001. AD, Alzheimer's disease; HNE, 4‐hydroxy‐nonenal; NT, 3‐nitrotyrosine; RFU, relative fluorescent unit.

Next, we examined changes in lipid peroxidation by brain region. First, we confirmed that like cortex samples, washed cerebellum only displayed a mild increase in heme comparing AD to control samples (Extended Data Figure 1A). Cerebellum samples did display increased HNE with AD compared to control, though to a lesser extent than in the cortex (Extended Data Figure 1B). In addition, while there was no difference in NT for the cerebellum between AD and cognitively normal, the AD cortex had increased NT (Extended Data Figure 1C). The most striking observation was the loss of association between *APOE* alleles and lipid peroxidation in the cerebellum (Extended Data Figure 1D), and the reversal of association of sex: males displayed higher levels of lipid peroxidation in AD samples, whereas no difference was seen in females in the cerebellum (Extended Data Figure 1E). Finally, to confirm that changes in lipid peroxidation correlate with “canonical” markers for AD pathology, we confirmed that our AD cortex and cerebellum samples display an increase in Aβ fibrils (Extended Data Figure 1F). Altogether, these data suggest that increased lipid peroxidation is associated with increased fibrillar amyloid. In addition, although the cerebellum demonstrates milder lipid peroxidation associated with AD pathology compared to the cortex, it is not completely devoid of pathology. Last, the association between lipid peroxidation and *APOE* status or sex is brain‐region specific.

### 3.3 Iron protein metabolism is decreased in AD

While washed brain tissues did not have increases in total iron, many studies have shown increased infiltration of blood cells into the brain during the progression of AD pathology due to microbleeds and loss of BBB integrity. For biochemical assays, it is important to remove blood to differentiate between the blood contributions versus brain tissue for iron and iron‐related proteins. Therefore, we next characterized the changes in iron metabolism in AD brains, which could be driven by blood cell infiltration. Brain cellular iron is imported by the divalent metal transporter 1 (DMT1) together with transferrin (TF) and its receptor TfR. The levels of TF and TfR differ by cell type.^31^ Heme iron is imported by heme carrier protein 1 (HCP1) and degraded by heme oxygenases (HMOX1 or HMOX2). Intracellular ferric iron is stored in complex with ferritin light chain (FTL) after oxidation of ferrous iron (Fe^2+^) by ferritin heavy chain (FTH1). The export of ferrous iron is mediated by ferroportin (FPN).

**FIGURE 2:**
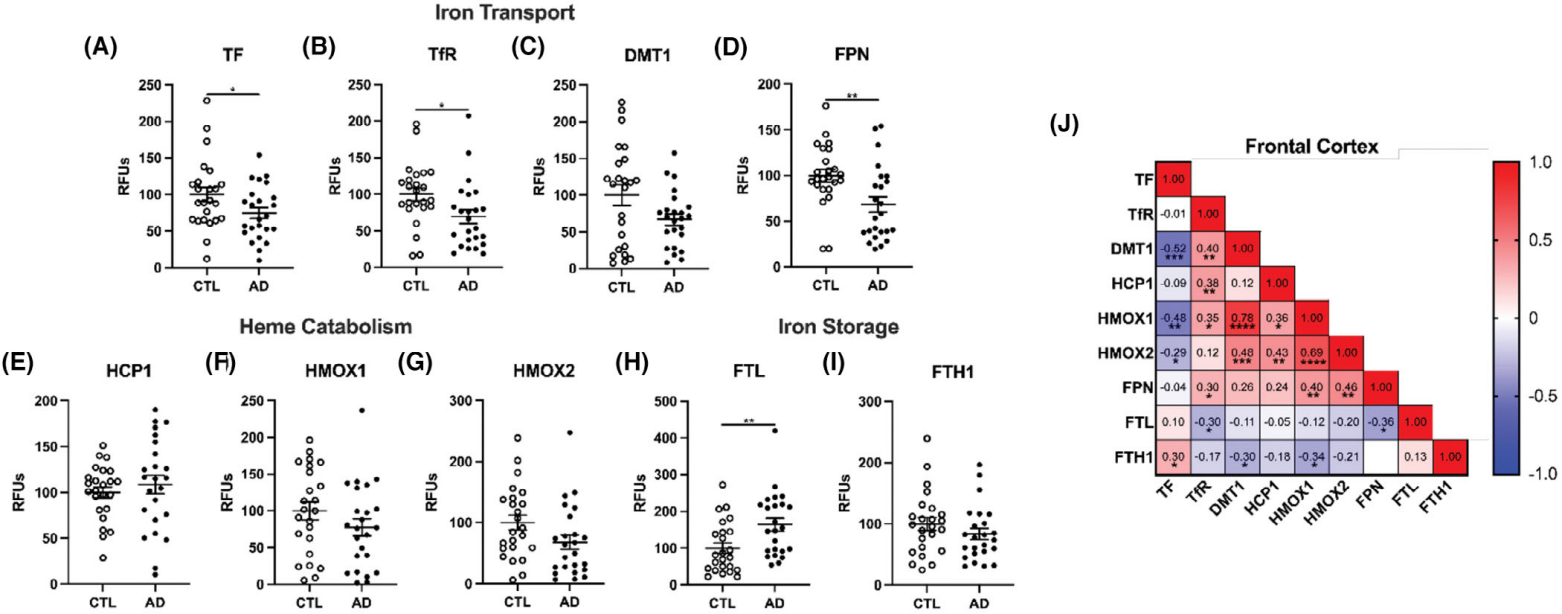
Iron transport and metabolism in AD prefrontal cortex (*n* = 24) compared to cognitively normal age‐matched controls (*n* = 24). Percent change from control as RFUs from Western blots: (A) TF, (B) TfR/CD71, (C) DMT1, (D) FPN, (E) HCP1, (F) HMOX1, (G) HMOX2, (H) FTL, (I) FTH1. J, Correlation matrix for iron metabolism in clinical AD. Significance, two‐tailed *t* test (A‐I), Spearman correlation (J): *p < 0.05, **p < 0.01, ***p < 0.001, ****p < 0.0001. AD, Alzheimer's disease; DMT1, divalent metal transporter 1; FPN, ferroportin; FTH1, ferritin heavy chain 1; FTL, ferritin light chain; HCP1, heme carrier protein 1; HMOX, heme oxygenase; RFU, relative fluorescent unit; TF, transferrin; TfR/CD71, transferrin receptor.

**EXTENDED DATA FIGURE 2 alz14541-fig-0010:**
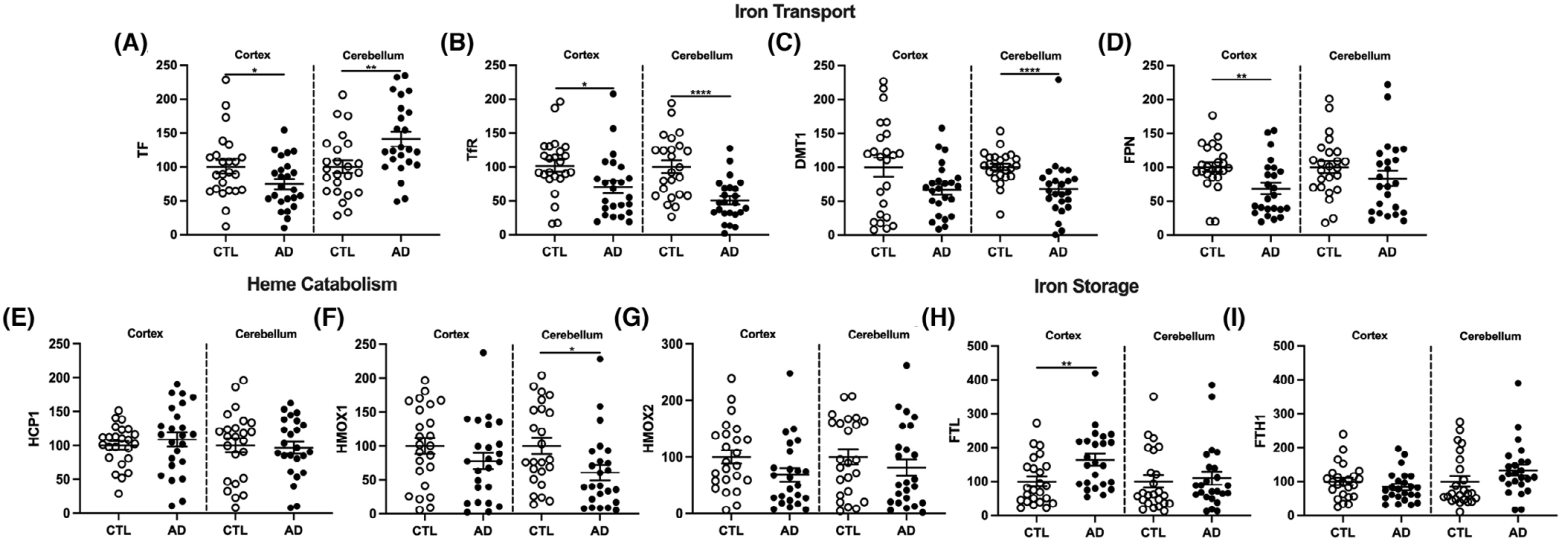
Iron transport and metabolism in AD versus cognitively normal prefrontal cortex and cerebellum. Western blots data as percent change from control as RFUs for (A) TF, (B) TfR/CD71, (C) DMT1, (D) FPN, (E), HCP1, (F) HMOX1, (G) HMOX2, (H) FTL, (I) FTH1. Significance, two‐tailed *t* test (A–I): ^*^ *p* < 0.05, ^**^ *p* < 0.01, ^****^ *p* < 0.0001. AD, Alzheimer's disease; DMT1, divalent metal transporter 1; FPN, ferroportin; FTH1, ferritin heavy chain 1; FTL, ferritin light chain; HCP1, heme carrier protein 1; HMX, heme oxygenase; RFU, relative fluorescent unit; TF, transferrin; TfR/CD71, transferrin receptor.

As expected, we saw that numerous proteins involved in iron were depleted in AD brains. First, iron‐transport proteins TF and its receptor TfR were decreased in AD brains compared to control (Figure 2A,B). Although we did not observe a statistically significant decrease in DMT1, there is a trend for a decrease in AD brains (Figure 2C), and FPN also showed significant reduction (Figure 2D). Interestingly, we did not observe major differences in heme catabolism (Figure 2E‐G). In addition, there was a increase in the iron storage protein, FTL (Figure 2H) with AD, though FTH1 did not show any difference between AD and control samples (Figure 2I). Relationships for iron import/export include negative correlations between iron storage protein FTL with iron import receptor TfR (*r* = –0.30, *P* = 0.04) and export protein by FPN (*r* = –0.36, *P* = 0.01; Figure 2J). *APOE* ε4 carriers had 2‐fold higher FTL than *APOE* ε3 in prefrontal cortex (Table S2 in supporting information). Interestingly, the AD cerebellum exhibited different changes in iron metabolism proteins compared to the cortex. The only iron transport protein that increased in the AD cerebellum compared to control samples was TF (Extended Data Figure 2A), with all others either displaying no change or a significant decrease (Extended Data Figure 2B‐D). Similar to most iron transport proteins, proteins involved in heme catabolism were also largely unchanged in the cerebellum, with only HMOX1 showing a slight decrease (Extended Data Figure 2E‐G). Finally, iron storage proteins also did not display any difference in the AD cerebellum (Extended Data Figure 2H‐I).

Overall, our data show that even though total iron levels do not change in the brain, there are significant differences in proteins related to iron metabolism. These changes seem to be primarily limited to the cortex, suggesting that the cortex is more susceptible to changes upon iron exposure compared to the cerebellum.

### 3.4 GSH synthesis is decreased with AD

**FIGURE 3:**
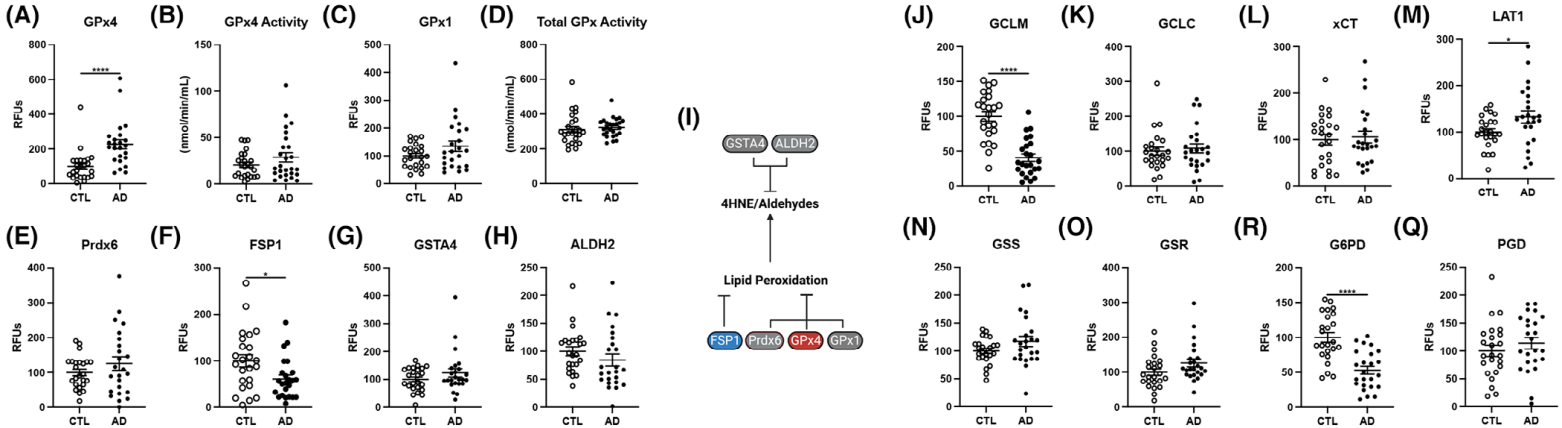
Enzymes that repair or mitigate lipid peroxidation and facilitate GSH production. Percent change from control as RFUs for human prefrontal cortex western blots and enzymatic activity in whole tissue lysate for (A) GPx4, (B) GPx4 activity, (C) GPx1, (D) total GPx activity, (E) Prdx6, (F) FSP1, (G) GSTA4, and (H) ALDH2. I, Schema of enzymatic repair of lipid peroxidation. GSH cycle enzyme levels by western blots: (J) GCLM, (K) GCLC, (L) xCT/SLC7A11, (M) LAT1, (N) GSS, (O) GSR, (P) G6PD, and (Q) PGD. Significance, two‐tailed *t* test (A‐H, J‐Q): *p < 0.05, ****p < 0.0001. ALDH2, aldehyde dehydrogenase 2; FSP1, ferroptosis suppressor protein 1; G6PD, glucose‐6‐phosphate dehyrdogenase; GCLC, glutamate‐cysteine ligase catalytic subunit; GCLM, glutamate‐cysteine ligase modifier subunit; GSH, glutathione; GSTA4, glutathione S‐transferase alpha 4; GPx, glutathione peroxidase; GSR, glutathione reductase; GSS, glutathione synthetase; PGD, phosphogluconate dehydrogenase; LAT1, L‐type amino acid transporter 1; PRdx6, peroxiredoxin 6; RFU, relative fluorescent unit.

Lipid peroxidation is driven by the loss of redox homeostasis. The tripeptide GSH conjugates to HNE for removal by several enzymes. GSH is also essential to reduce peroxides by the GPx enzyme and Prdx families. GPx4 and Prdx6 are the only known lipid hydroperoxidases capable of reducing oxidized phospholipids. These enzymes are critical in safeguarding against lipid peroxidation and ferroptosis. All other GPx family members also detoxify hydroperoxides using GSH, with GPx1 being the most abundant GPx family member in the brain.^57^ Shockingly, we saw an increase in GPx4 levels in AD prefrontal cortex compared to controls (Figure 3A). However, total GPx4 activity was not changed (Figure 3B), suggesting that although enzyme levels may be higher, the functional output of these hydroperoxidases are unchanged. In addition, GPx1 levels and activity, as well as levels of Prdx6, were unchanged in the AD cortex (Figure 3C‐E). Finally, ferroptosis suppressor protein 1 (FSP1), which also protects against lipid peroxidation through the quinol cycle in a GSH‐independent manner, did show a significant reduction in the AD cortex (Figure 3F).

To address the discrepancy between increased lipid peroxidation observed in AD brains and the lack of changes seen in detoxification enzymes, we next sought to determine whether there were defects in enzymes involved in GSH synthesis (Figure 3I). The glutathione S‐transferases (GST) conjugates GSH with diverse metabolites and xenobiotics. Biosynthesis of GSH depends on cystine import via the proteins xCT (SLC7A11) and L‐type amino acid transporter 1 (LAT1; SLC3A2/CD98). Imported cystine is rapidly reduced to cysteine by the intracellular reducing environment.^58^ The rate‐limiting step in GSH synthesis^59^ is the formation of γ‐glutamylcysteine, catalyzed by glutamate‐cysteine ligase, with two subunits GCLC (catalytic) and GCLM (modulatory); the binding of GCLM to GCLC accelerates GSH synthesis 4‐fold. Next, GSH forms from the addition of glycine to γ‐glutamylcysteine by glutathione synthetase (GSS). Because GPx4 and Prdx6 activities are dependent on GSH, a decreased total pool of GSH would also increase lipid peroxidation, even if GPx4 and Prdx6 protein levels remain unchanged. GSH itself could not be measured due to its rapid oxidation upon death and associated artifacts, which is impossible to prevent in human *post mortem* tissue.^60^ In the prefrontal cortex, the glutamate–cysteine ligase subunits were differentially altered by AD: the GCLM modulatory subunit was 60% lower, while GCLC in the catalytic subunit was unaltered (Figure 3J,K). LAT1 (L‐type amino acid importer transporter) increased by 33% in AD, while xCT did not differ, contrary to the 20% increase reported by Ashraf et al.^8^ (Figure 3L,M). Two enzymes of the GSH cycle were not altered in the AD prefrontal cortex: GSS, the final step in GSH synthesis (Figure 3N), and glutathione reductase (GSR) (Figure 3O), which uses nicotinamide adenine dinucleotide phosphate (NADPH) to reduce glutathione disulfide back to GSH.

Related metabolic cycles also showed selective AD changes. For the pentose phosphate pathway, glucose‐6‐phosphate dehyrdogenase (G6PD) was decreased by 45% in AD relative to controls, while phosphogluconate dehydrogenase (PGD) was unchanged (Figure 3P,Q). These enzymes oxidize glucose to generate NAPDH required by GSR for oxidized GSH reduction (GSSG). Two groups of enzymes had correlated levels: G6PD and PGD for NADPH production were positively correlated with GSR (both *r* = 0.31, *P* = 0.03), as were the amino acid importer LAT1 and cystine importer xCT (*r* = 0.49, *P* < 0.001; not shown). Collectively, the decreases in GCLM and G6PD in the AD prefrontal cortex suggest reduced GSH levels, which contributes to the increased lipid peroxidation, despite a lack of change in GPx4 or antioxidant enzymes.

**EXTENDED DATA FIGURE 3 alz14541-fig-0011:**
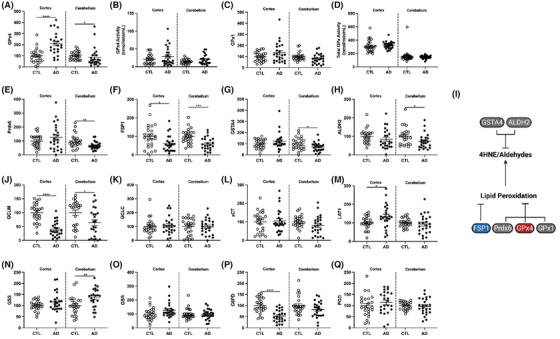
Enzymes that mitigate lipid peroxidation and facilitate GSH production. Western blot as percent change from control as RFUs or enzyme activity units for prefrontal cortex or cerebellum from whole tissue lysate for (A) GPx4, (B) GPx4 activity, (C) GPx1, (D) total GPx activity, (E) Prdx6, (F) FSP1, (G) GSTA4, and (H) ALDH2. I, Enzymatic repair of lipid peroxidation. GSH cycle enzyme levels by western blots, as RFUs: (J) GCLM, (K) GCLC, (L) xCT/SLC7A11, (M) LAT1, (N) GSS, (O) GSR, (P) G6PD, and (Q) PGD. Significance, two‐tailed *t* test (A–H,J–Q): ^*^ *p* < 0.05, ^**^ *p* < 0.01, ^***^ *p* < 0.001, ^****^ *p* < 0.0001. ALDH2, aldehyde dehydrogenase 2; FSP1, ferroptosis suppressor protein 1; G6PD, glucose‐6‐phosphate dehyrdogenase; GCLC, glutamate‐cysteine ligase catalytic subunit; GCLM, glutamate‐cysteine ligase modifier subunit; GPx, glutathione peroxidase; GSH, glutathione; GSR, glutathione reductase; GSS, glutathione synthetase; GSTA4, glutathione S‐transferase alpha 4; LAT1, L‐type amino acid transporter 1; PGD, phosphogluconate dehydrogenase; PRdx6, peroxiredoxin 6; RFU, relative fluorescent unit.

Unlike the prefrontal cortex, the AD cerebellum showed more changes to lipid hydroperoxidases. GPx4 levels decreased in AD cerebellum (Extended Data Figure 3A), although total activity of GPx4 was unchanged (Extended Data Figure 3B). In addition, although GPx1 levels and activity were unchanged like AD cortex (Extended Data Figure 3C‐D), Prdx6 levels were lower in AD cerebellum (Extended Data Figure 3E). Finally, consistent with changes in AD cortex, FSP1 levels were also decreased in AD cerebellum (Extended Data Figure 3F). Overall, AD cerebellum displayed more changes in antioxidant enzymes compared to the cortex, in addition to showing a similar decrease in the GSH synthesis enzyme GCLM (Extended Data Figure 3J). Other GSH synthesis enzymes were largely unchanged, apart from GSS, which increased in the cerebellum (Extended Data Figure 3K‐Q).

### 3.5 Lipid peroxidation in LRs is increased and antioxidant defense is diminished in AD

**FIGURE 4:**
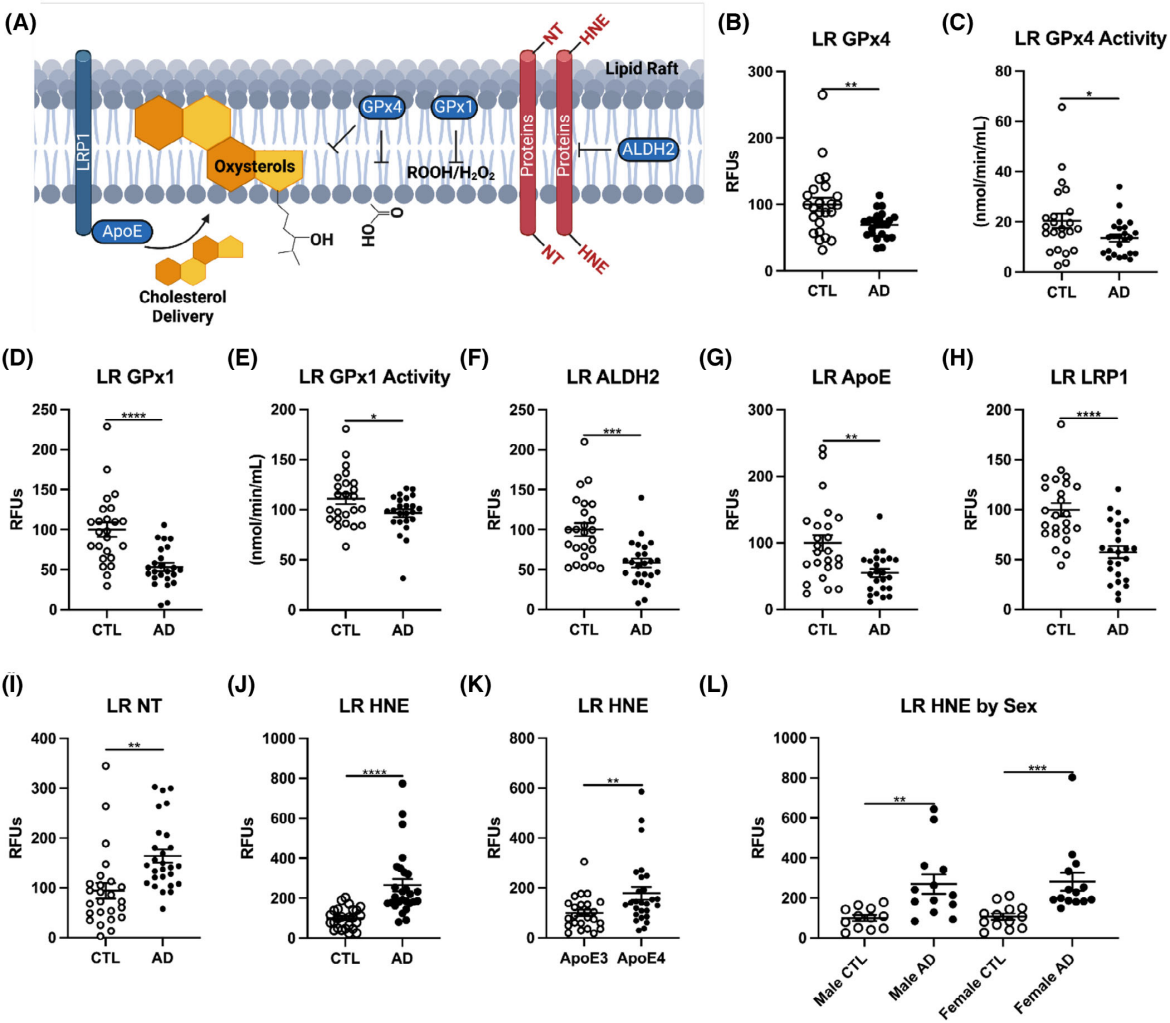
The lipid raft membrane fraction has increased damage and reduced antioxidant defense during AD. A, Schema of lipid raft damage, protective mechanisms, and cholesterol shuttling. Percent change from control as RFUs for western blot data and enzymatic activity (B) GPx4, (C) GPx4 activity, (D) GPx1, (E) GPx1 activity, (F) ALDH2, (G) *APOE*, (H) LRP1. Dot blots for (I) NT and HNE presented as (J) CTL versus AD, (K) *APOE* allele, and (L) sex. Significance, by two‐tailed *t* test (B‐K) or one‐way analysis of variance with Tukey post hoc (L): ^*^ *p* < 0.01, ^**^ *p* < 0.01, ^***^ *p* < 0.001, ^****^ *p* < 0.0001. AD, Alxheimer's disease; ALDH2, aldehyde dehydrogenase 2; *APOE*, apolipoprotein E; CTL, normal control; GPx, glutathione peroxidase; HNE, 4‐hydroxy‐nonenal; LRP1, low‐density lipoprotein receptor‐related protein 1; NT, 3‐nitrotyrosine; RFU, relative fluorescent unit.

Because measurements of whole‐cell protein levels of antioxidant defense enzymes were inconclusive, we next examined the local environment of subcellular LRs (Figure 4A), the hub of signal transduction, and the site of APP processing. LR isolations were verified and compared to traditional ultracentrifugation methods.^27^ First, we identified which of the eight GPx isoforms were present in LRs and only detected GPx4 and GPx1 (Figure S4 in supporting information). Prdx6 was not detected. AD LRs had significantly lower levels of the membrane protective enzymes GPx4 and GPx1, as well as decreased activity of both GPx4 and GPx1 (Figure 4B‐E). In addition, we observed lower levels of aldehyde dehydrogenase 2 (ALDH2), which clears free HNE and the Aβ clearance proteins, *APOE*, and low‐density lipoprotein receptor‐related protein 1 (LRP1) in AD LRs (Figure 4F‐H). Importantly, the decreases in antioxidant protection and activity in the AD cortex were further substantiated by increased LR oxidation from HNE and NT (Figure 4I‐J), paralleling increases in total tissue (Figure 1B‐E). This increase in lipid peroxidation is associated with the *APOE* ε4 allele, which exhibited higher levels of HNE in LRs (Figure 4K), but no associations with sex were identified, as both males and females showed similar increases (Figure 4L). Overall, these data suggest that measurements of oxidative stress, particularly linking changes to antioxidant defense and lipid peroxidation, are most robust in the LRs for AD samples. Collectively, our data strongly correlate the loss of antioxidant enzymes in rafts with lipid oxidative damage in the AD cortex.

**EXTENDED DATA FIGURE 4 alz14541-fig-0012:**
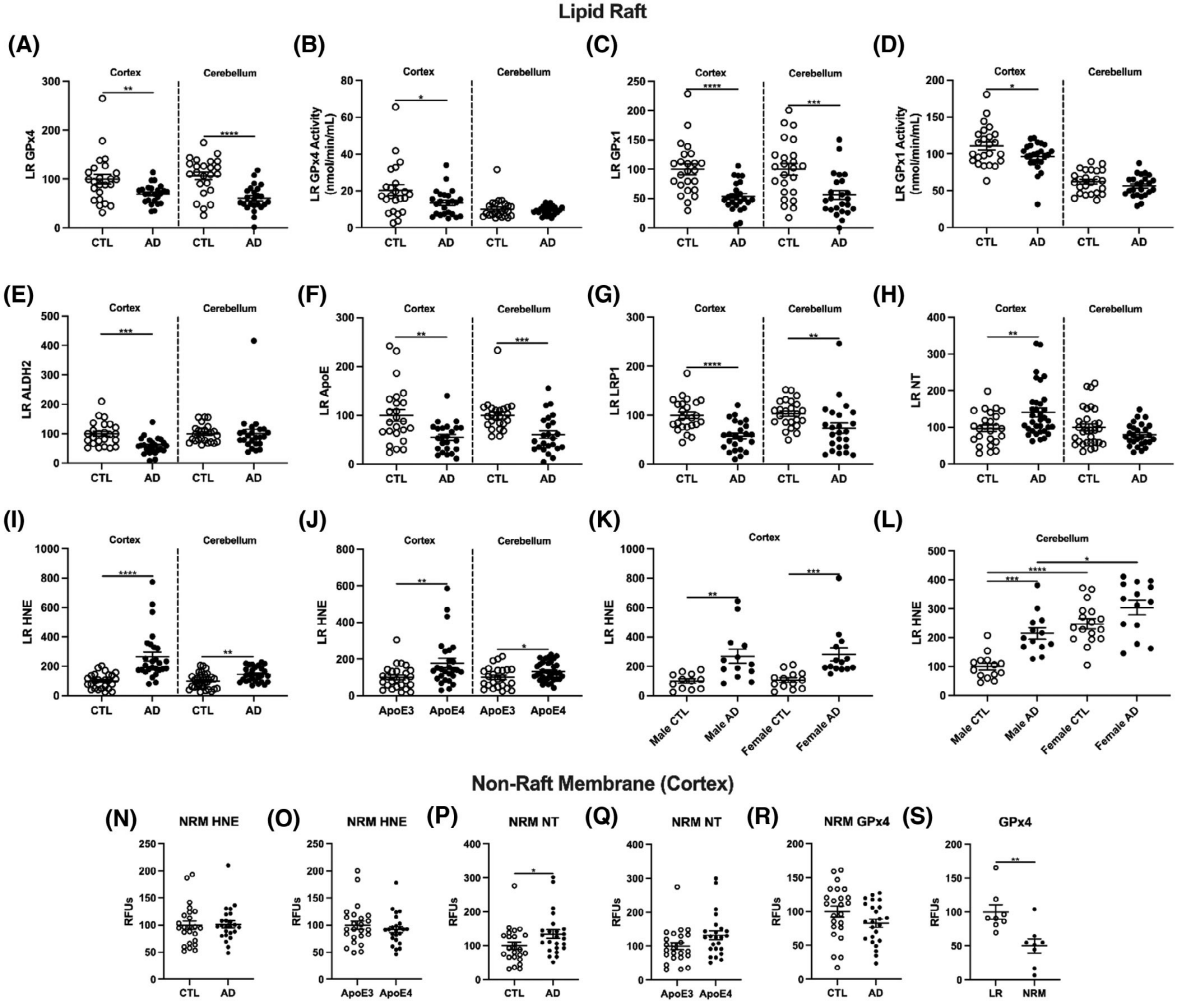
The AD lipid raft has increased damage and reduced antioxidant defense. A, Schema of lipid raft damage, protective mechanisms, and cholesterol shuttling in AD prefrontal cortex and cerebellum. Western blots as percent change from control as RFUs or enzymatic activity: (B) GPx4, (C) GPx4 activity, (D) GPx1, (E) GPx1 activity, (F) ALDH2, (G) *APOE*, (H) LRP1. Dot blots for (I) NT and HNE presented as (J) CTL versus AD, (K) *APOE* allele, and sex for (L) cortex and (M) cerebellum. Significance, two‐tailed *t* test (A–J, N–S), one‐way analysis of variance with Tukey post hoc test (K,L): ^*^ *p* < 0.05, ^**^ *p* < 0.01, ^***^ *p* < 0.001, ^****^ *p* < 0.0001. AD, Alzheimer's disease; ALDH2, aldehyde dehydrogenase 2; *APOE*, apolipoprotein E; CTL, normal control; GPx, glutathione peroxidase; HNE, 4‐hydroxy‐nonenal; LRP1, low‐density lipoprotein receptor‐related protein 1; NT, 3‐nitrotyrosine; RFU, relative fluorescent unit.

Next, we performed similar LR measurements in the cerebellum and found that like the AD cortex, the AD cerebellum exhibited a significant decrease in GPx4 and GPx1 levels. However, unlike the cortex, GPx4 and GPx1 activities were not decreased in the AD cerebellum (Extended Data Figure 4A‐E). Although ALDH2 levels were unchanged, *APOE* and LRP1 were significantly decreased in the AD cerebellum (Extended Data Figure 4F‐H). Similar to what was observed in whole‐cell lipid peroxidation, AD cerebellum LRs displayed no change in NT, but higher levels of HNE, which is consistent with our previous conclusion that concerning lipid peroxidation, the cerebellum samples display lower AD pathology than the prefrontal cortex. Similar to the cortex, the cerebellum also showed higher lipid peroxidation in *APOE* ε4 carriers (Extended Data Figure 4L) with no observable difference between males and females (Extended Data Figure 4M).

The NRM fraction from these same samples was also examined, which unexpectedly showed increased oxidative damage for NT but not HNE (Extended Data Figure 4N‐Q). No *APOE* allele differences were observed for the NRM. NRM GPx4 did not differ by AD (Extended Data Figure 4R). GPx4 in non‐membrane fraction was 50% lower below LR from the same tissues (Extended Data Figure 4S), suggesting more localization of GPx4 to LRs. These data suggest some membrane domains are selectively vulnerable to oxidation.

#### 3.5.1 Experimental iron chelation improves pathology in AD mouse models

Our data provide strong correlative evidence that dysfunction of iron homeostasis can drive lipid peroxidation, which is exacerbated in LRs due to a loss of antioxidant enzymes in AD brains. However, one major limitation of studies with *post mortem* brain tissue is the inability to experimentally test causality. Therefore, we sought to evaluate the impact of the removal of iron by chelation using DFO on AD pathology in the early‐onset familial AD (EFAD) mouse model. DFO was chosen over other chelators because of its use in AD patients^40^ and experimental studies with [H^3^]DFO showing it crossed the BBB, yielding 3‐fold higher levels per gram in the brain than blood and kidney.^61^ We further validated this by performing magnetic resonance imaging on wild‐type mice, showing decreases in brain iron with acute DFO treatment.^62^ In our experimental paradigm, female EFAD mice received DFO in two modes: by lab chow for 2 weeks or by intraperitoneal injection (IP) twice daily for 1 week (see extended data for comparison of DFO modes).

**FIGURE 5:**
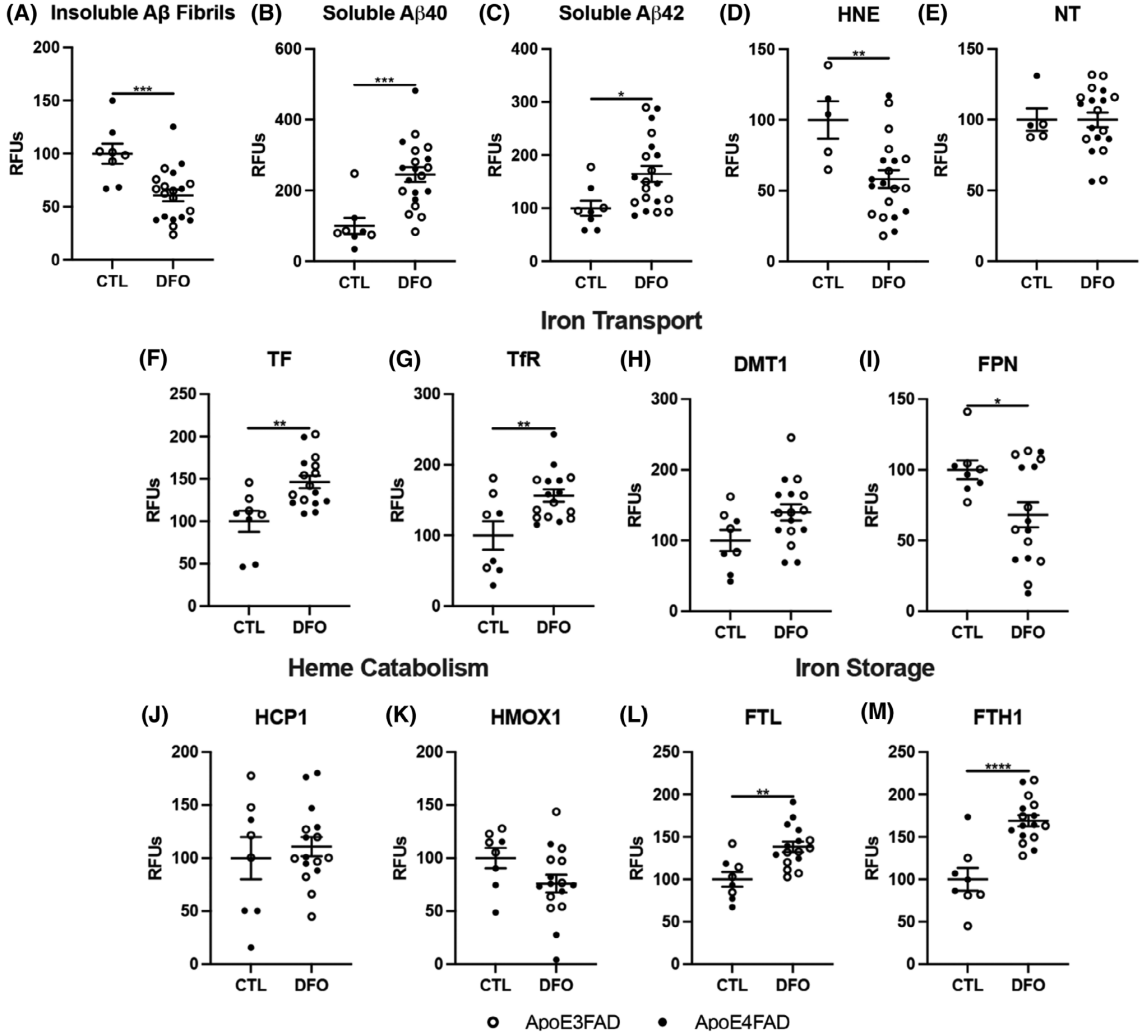
DFO chelation of EFAD mouse cortex for amyloid and iron. Percent change from control as RFUs for (A) insoluble Aβ fibrils, (B) soluble Aβ40, (C) soluble Aβ42, (D) HNE, (E) NT, (F) TF, (G) TfR, (H) DMT1, (I) FPN, (J) HCP1, (K) HMOX1, (L) FTL, and (M) FTH1. Significance, two‐tailed *t* test: ^*^ *p* < 0.05, ^**^ *p* < 0.01, ^***^ *p* < 0.001, ^****^ *p* < 0.0001. Aβ, amyloid beta; DFO, deferoxamine; DMT1, divalent metal transporter 1; EFAD, early‐onset familial Alzheimer's disease; FPN, ferroportin; FTH1, ferritin heavy chain 1; FTL, ferritin light chain; HCP1, heme carrier protein 1; HMOX, heme oxygenase; HNE, 4‐hydroxy‐nonenal; NT, 3‐nitrotyrosine; TF, transferrin; TfR, transferrin receptor.

**EXTENDED DATA FIGURE 5 alz14541-fig-0013:**
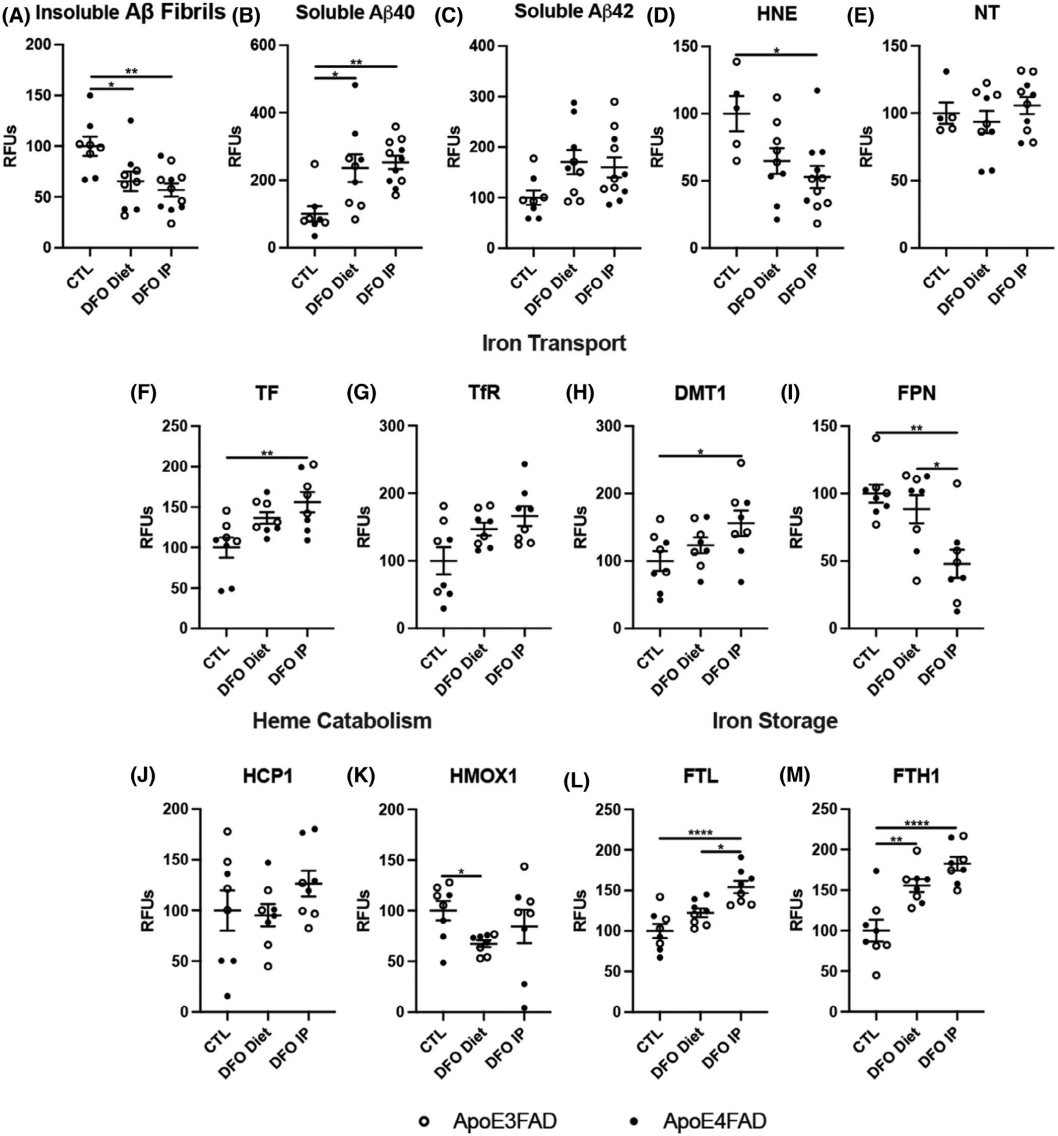
DFO chelation of EFAD mouse cortex comparing diet and IP for amyloid and iron proteins. Percent change from control as RFUs for (A) insoluble Aβ fibrils, (B) soluble Aβ40, (C) soluble Aβ42, (D) HNE, (E) NT, (F) TF, (G) TfR, (H) DMT1, (I) FPN, (J) HCP1, (K) HMOX1, (L) FTL, and (M) FTH1. Significance, two‐tailed *t* test: ^*^ *p* < 0.05, ^**^ *p* < 0.01, ^***^ *p* < 0.001, ^****^ *p* < 0.0001. Aβ, amyloid beta; DFO, deferoxamine; DMT1, divalent metal transporter 1; EFAD, early‐onset familial Alzheimer's disease; FPN, ferroportin; FTH1, ferritin heavy chain 1; FTL, ferritin light chain; HCP1, heme carrier protein 1; HMOX, heme oxygenase; HNE, 4‐hydroxy‐nonenal; IP, intraperitoneal injection; NT, 3‐nitrotyrosine; RFU, relative fluorescent unit; TF, transferrin; TfR/CD71, transferrin receptor.

First, we confirmed that DFO treatment ameliorated canonical markers of AD: specifically, we found that DFO‐treated mice displayed a significant reduction in fibrillar Aβ (Figure 5A, Extended Data Figure 5A), and a significant increase in soluble Aβ peptides (Figure 5B,C, Extended Data Figure 5B,C). Importantly, we observed a significant reduction in lipid peroxidation by measuring HNE in DFO‐treated mice, and no change in NT, further confirming the role of iron in lipid peroxidation (Figure 5D,E, Extended Data Figure 5D,E). To determine whether these changes were due to changes in iron handling, we next measured iron metabolism proteins. We found that many iron transport proteins and iron storage proteins were significantly increased, although no significant changes were seen in heme catabolism proteins with DFO treatment (Figure 5F‐M, Extended Data Figure 5F‐M).

**FIGURE 6:**
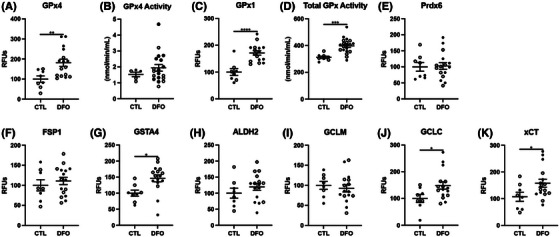
DFO chelation of EFAD mouse cortex for antioxidant proteins. Percent change from control as RFUs or enzymatic activity for (A) GPx4, (B) GPx4 activity, (C) GPx1, (D) GPx1 activity, (E) Prdx6, (F) FSP1, (G) GSTA4, (H) ALDH2, (I) GCLM, (J) GCLC, (K) xCT. Significance, two‐tailed *t* test: ^*^ *p* < 0.05, ^**^ *p* < 0.01, ^***^ *p* < 0.001, ^****^ *p* < 0.0001. ALDH2, aldehyde dehydrogenase 2; DFO, deferoxamine; EFAD, early‐onset familial Alzheimer's disease; FSP1, ferroptosis suppressor protein 1; GCLC, glutamate‐cysteine ligase catalytic subunit; GCLM, glutamate‐cysteine ligase modifier subunit; GPx, glutathione peroxidase; GSTA4, glutathione S‐transferase alpha 4; PRdx6, peroxiredoxin 6; RFU, relative fluorescent unit.

**EXTENDED DATA FIGURE 6 alz14541-fig-0014:**
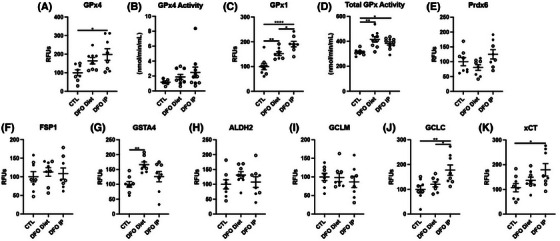
DFO chelation of EFAD mouse cortex comparing diet and IP for antioxidants. Percent change from control as RFUs for (A) GPx4, (B) GPx4 activity, (C) GPx1, (D) GPx1 activity, (E) Prdx6, (F) FSP1, (G) GSTA4, (H) ALDH2, (I) GCLM, (J) GCLC, (K) xCT. Significance, one‐way analysis of variance with Tukey post hoc test (A–K): ^*^ *p* < 0.05, ^**^ *p* < 0.01, ^***^ *p* < 0.001, ^****^ *p* < 0.0001. ALDH2, aldehyde dehydrogenase 2; DFO, deferoxamine; EFAD, early‐onset familial Alzheimer's disease; FSP1, ferroptosis suppressor protein 1; GCLC, glutamate‐cysteine ligase catalytic subunit; GCLM, glutamate‐cysteine ligase modifier subunit; GSTA4, glutathione S‐transferase alpha 4; PRdx6, peroxiredoxin 6; RFU, relative fluorescent unit.

As previously mentioned, because ferroptosis is induced by a combination of iron overload and/or an increase in oxidative damage, we next measured whether iron chelation through DFO was sufficient to improve redox handling and antioxidant activity in the brain. DFO treatment resulted in a significant increase in GPx4 and GPx1 levels, with a significant increase in GPx1 activity, but not GPx4 activity, although a trend of increase was seen for GPx4 activity in some animals (Figure 6A‐D, Extended Data Figure 6A‐D). In addition, glutathione S‐transferase alpha 4 (GSTA4), GCLC, and xCT showed a significant increase in DFO‐treated animals, although several others (Pdrx6, FSP1, ALDH2, GCLM) showed no difference (Figure 6E‐K, Extended Data Figure 6E‐K). Overall, these experimental studies provide direct evidence that the removal of iron and improved iron handling can reduce oxidative damage, improve antioxidant defense, and ameliorate AD pathology in mice.

#### 3.5.2 AD pathogenesis downstream of iron‐related oxidative damage is driven by changes in Nrf2 signaling

To determine whether the outcome of iron‐related oxidative damage in AD pathology was due to a dysregulation of iron homeostasis directly or through changes in downstream antioxidant pathways, we next measured changes in major transcriptional regulators of iron and oxidative damage handling. Interestingly, we found no major differences in the transcription factors iron regulatory protein (IRP)1 and IRP2, involved in the regulation of iron genes in the AD cortex, although a decrease in IRP2 was observed in the AD cerebellum (Figure S5A‐C in supporting information). Importantly, we saw a significant decrease in Nrf2 in the AD cortex, one of the major transcription factors involved in antioxidant defense pathways and iron regulation (Figure S5D). Interestingly, the AD cerebellum did not display a significant decrease in Nrf2. The AD cortex also showed higher levels of nuclear factor kappa B (NF‐kB), associated with inflammation known to be elevated during AD (Figure S5E). Like Nrf2, NF‐kB did not show any observable differences in the cerebellum. Finally, there was a significant increase in nuclear receptor coactivator 4 (NCOA4) in the AD cortex (Figure S5F), which signals iron degradation by ferritinophagy.^63^ These data were consistent with our other observations that changes in antioxidant defense pathways were more pronounced than changes in iron metabolism in the AD brain. Moreover, consistent with our previous data, the AD cortex displayed more oxidative damage than the cerebellum.

To determine whether this depletion of nuclear Nrf2 correlated with changes in gene expression of genes involved in antioxidant defense, we performed RNA‐seq analysis on control‐matched and case‐matched AD cortex and cerebellum (Figure S5G‐H). Although for this study, data were pooled into AD versus control, we ensured that RNA‐seq analysis was performed across males, females, and *APOE* genotypes, which can be analyzed across sexes and genotypes in a future study. Gene Ontology (GO) analysis revealed that many differentially expressed genes in the AD samples were those associated with numerous pathways previously implicated in AD pathology, including mitochondrial dysfunction (Figure S5I). Many of these significant GO terms overlapped between the cortex and the cerebellum, although several terms were not shared, suggesting again that the cerebellum is not fully AD resistant, although the cortex displays signs of higher pathology. As expected, we did not observe major differences in gene expression of iron‐related genes. More importantly, we found depletion of several Nrf2 targets in the AD cortex, but not in the cerebellum (Figure S5J), which directly correlates with the decrease in nuclear Nrf2 found specifically in the AD cortex. Transcriptome changes shared only nine common DEGs between brain regions. CD163, the hemoglobin–haptoglobin scavenger that increases in response to microbleeds, was shared between brain regions with AD and was among the top responding mRNAs.^64^ Other genes previously associated with AD or dementia were differentially expressed in both brain regions: *CHI3L2, FCGBP, GLI1, STAB1, APOC1, MYO7A*, and long non‐coding RNA JHDM1D‐AS1 (Figure S5K). Taken together, our assay of transcriptional pathways supports our current hypothesis that iron toxicity is directly associated with a loss of antioxidant defense in AD pathology.

Consistent with the human data, we also found that DFO treatment of EFAD mice did not increase IRP or NOCA4 but did result in a significant increase in Nrf2 (Figure S5L‐O), again suggesting that most pathogenic pathways downstream of iron are through changes in antioxidant defense pathways.

### 3.6 Neuronal loss in AD is associated with increased ferritin and lipid peroxidation, and diminished antioxidant defenses

**FIGURE 7:**
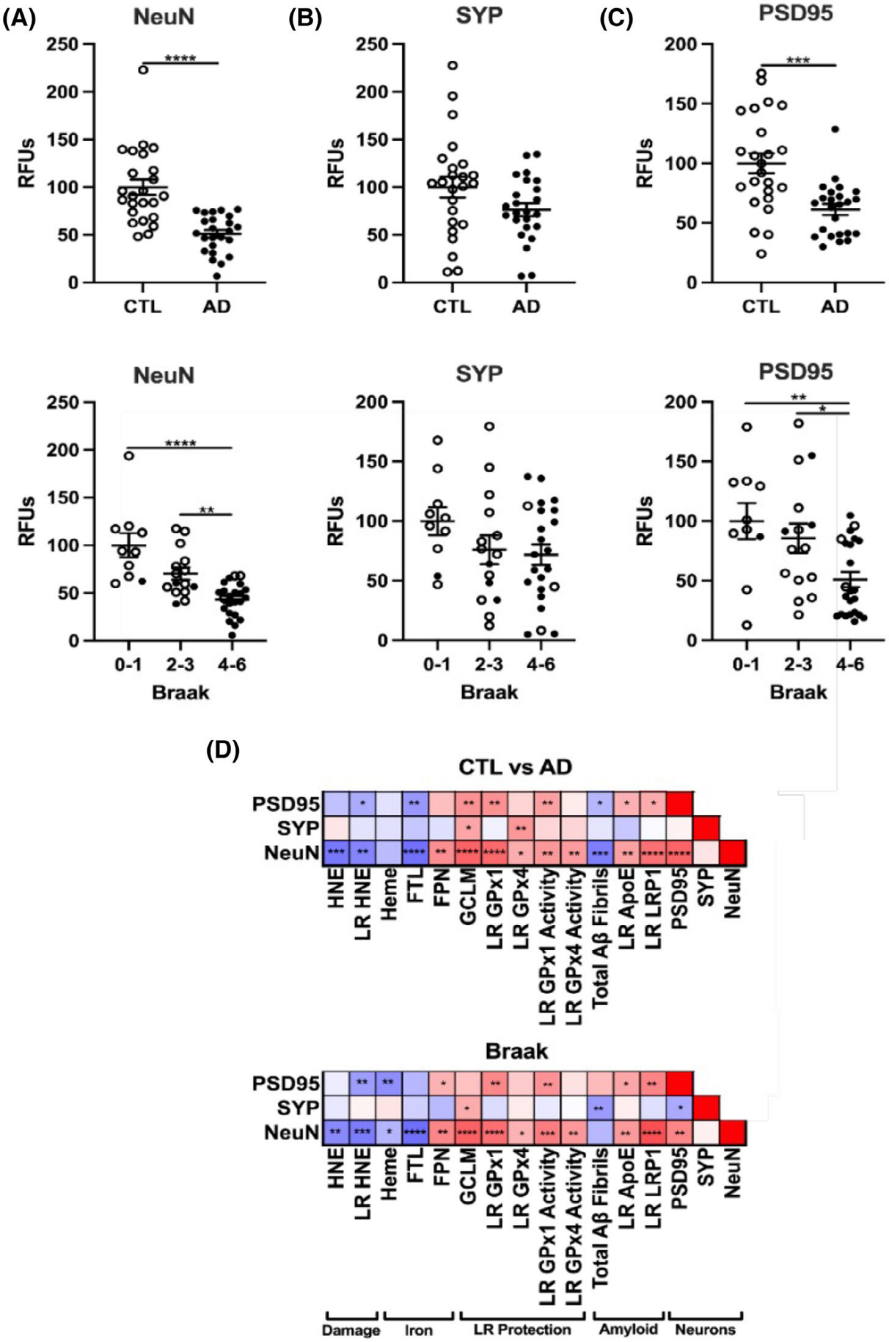
Neuronal loss and transcriptional changes in AD prefrontal cortex. Western blots percent change from control as RFUs of neuronal markers by CTL versus AD, or Braak stage.^27^ A, Neuronal nuclei antigen (NeuN), (B) synaptophysin 1 (SYP), (C) postsynaptic density protein 95 (PSD95). D, Correlation matrix for variables with significant relationships to NeuN by CTL versus AD or Braak. Significance, two‐tailed *t* test (CTL vs. AD), one‐way analysis of variance (Braak) with Tukey post hoc, or Spearman correlation: ^*^ *p* < 0.05, ^**^ *p* < 0.01, ^***^ *p* < 0.001, ^****^ *p* < 0.0001. AD, Alzheimer's disease; CTL, normal control; RFU, relative fluorescent unit.

**EXTENDED DATA FIGURE 7 alz14541-fig-0015:**
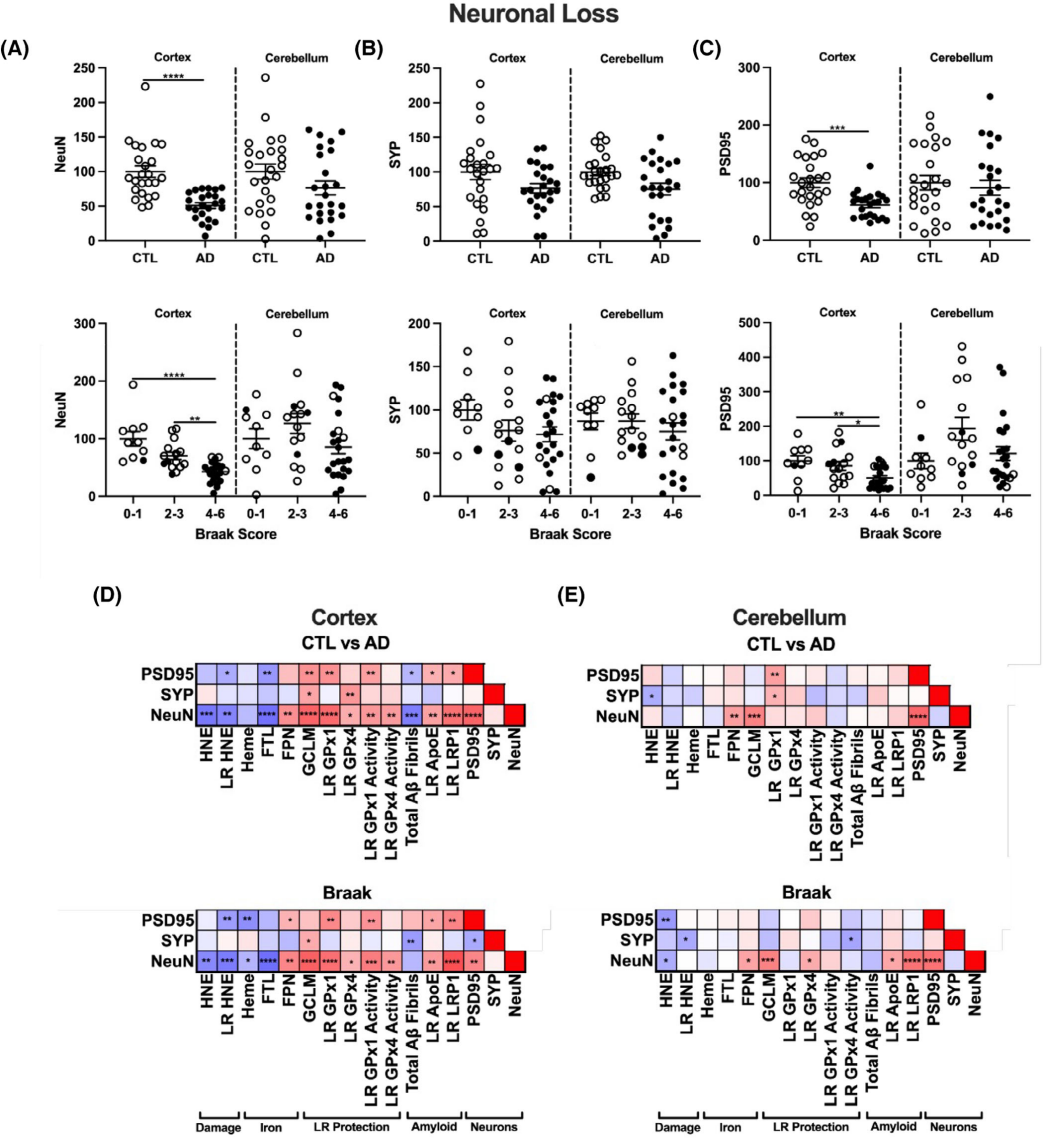
Neuronal loss in AD. Western blots as percent change from control RFUs of neuronal markers shown by clinical CTL versus AD or Braak stage.^27^ A, neuronal nuclei antigen (NeuN), (B) synaptophysin 1 (SYP), (C) postsynaptic density protein 95 (PSD95). Correlation matrixes for variables with significant relationships to NeuN for (D) prefrontal cortex and (E) cerebellum. Significance by two‐tailed *t* test (CTL vs. AD), one‐way analysis of variance (Braak) with Tukey post hoc, or by Spearman correlation: ^*^ *p* < 0.05, ^**^ *p* < 0.01, ^***^ *p* < 0.001, ^****^ *p* < 0.0001. AD, Alzheimer's disease; CTL, normal control; RFU, relative fluorescent unit.

To determine the physiological consequence of iron‐mediated oxidative damage to lipids, we then measured neuronal loss in AD samples. While neuronal loss dramatically increases with pathology in AD, the relationship between neuronal loss iron or membrane damage in AD is still lacking. Therefore, we analyzed bulk neuronal content in the prefrontal cortex and cerebellum using NeuN (neuronal nuclear antigen), presynaptic SYP (synaptophysin1), and postsynaptic PSD95 (postsynaptic density protein 95). Strikingly, we saw a significant depletion of NeuN and PSD95, and a trend for a decrease in SYP in the AD cortex compared to cognitively normal (Figure 7A‐C), although no significant changes were seen for any markers in the cerebellum (Extended Data Figure 7A‐C). These data were also stratified by Braak score and show a similar trend, whereby higher Braak scores correlated with lower NeuN and PSD95 in the cortex, but not the cerebellum (Figure 7D, Extended Data Figure 7D,E). In addition, neuronal markers varied inversely with cortex tissue levels of heme by Braak score, while NeuN varied inversely with ferritin light chain (Figure 7D). The relationship for NeuN and heme differed by Braak stage, but not by clinical dementia status. These inverse associations provide the first direct associations between brain iron markers and brain region‐specific neuron loss during AD. The inverse correlation of NeuN with HNE strengthens the relationship of lipid peroxidation to neuron loss. Many of these relationships were weakened or absent from the cerebellum, consistent with its reduced neuronal loss (Extended Data Figure 7D,E). However, positive correlations between NeuN and FPN or NeuN and GCLM protein levels were the only shared relationships by both criteria and in the prefrontal cortex and cerebellum. These relationships highlight the role of iron export by FPN or GSH production by GCLM for neuronal survival. Overall, these data provide direct evidence that the physiological consequence of increased iron‐related oxidative damage results in neuronal cell death, likely through the induction of ferroptosis.

### 3.7 *APOE* allele associations with LRs, lipid peroxidation, and antioxidant defense

**FIGURE 8:**
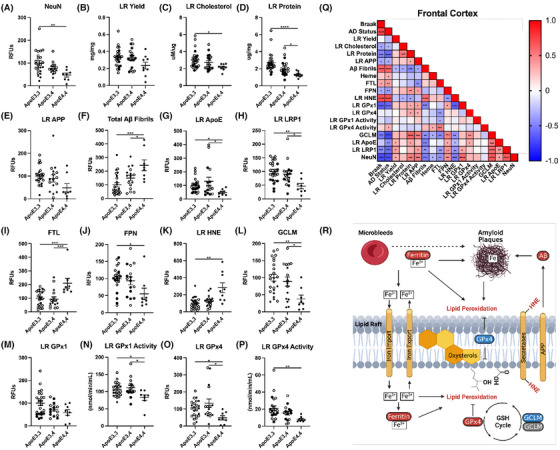
*APOE* allele associations with lipid rafts, antioxidant defense, and amyloid in the human prefrontal cortex: (A) NeuN, (B) LR yield, (C) LR cholesterol, (D) total LR protein, (E) LR APP, (F) total Aβ fibrils, (G) LR *APOE*, (H) LR LRP1, (I) FTL, (J) FPN, (K) LR HNE, (L) GCLM, (M) LR GPx1, (N) LR GPx1 activity, (O) LR GPx4, (P) LR GPx4 activity. Q, Correlation matrix by *APOE* allele. One‐way analysis of variance by Tukey post hoc, or Spearman correlation: ^*^ *p* < 0.05, ^**^ *p* < 0.01, ^***^ *p* < 0.001, ^****^ *p* < 0.0001. R, Model for ferroptosis in AD. Aβ, amyloid beta; AD, Alzheimer's disease; *APOE*, apolipoprotein E; APP, amyloid precursor protein; FPN, ferroportin; FTL, ferritin light chain; GCLM, glutamate‐cysteine ligase modifier subunit; GPx, glutathione peroxidase; HNE, 4‐hydroxy‐nonenal; LR, lipid raft; LRP1, low‐density lipoprotein receptor‐related protein 1.

**EXTENDED DATA FIGURE 8 alz14541-fig-0016:**
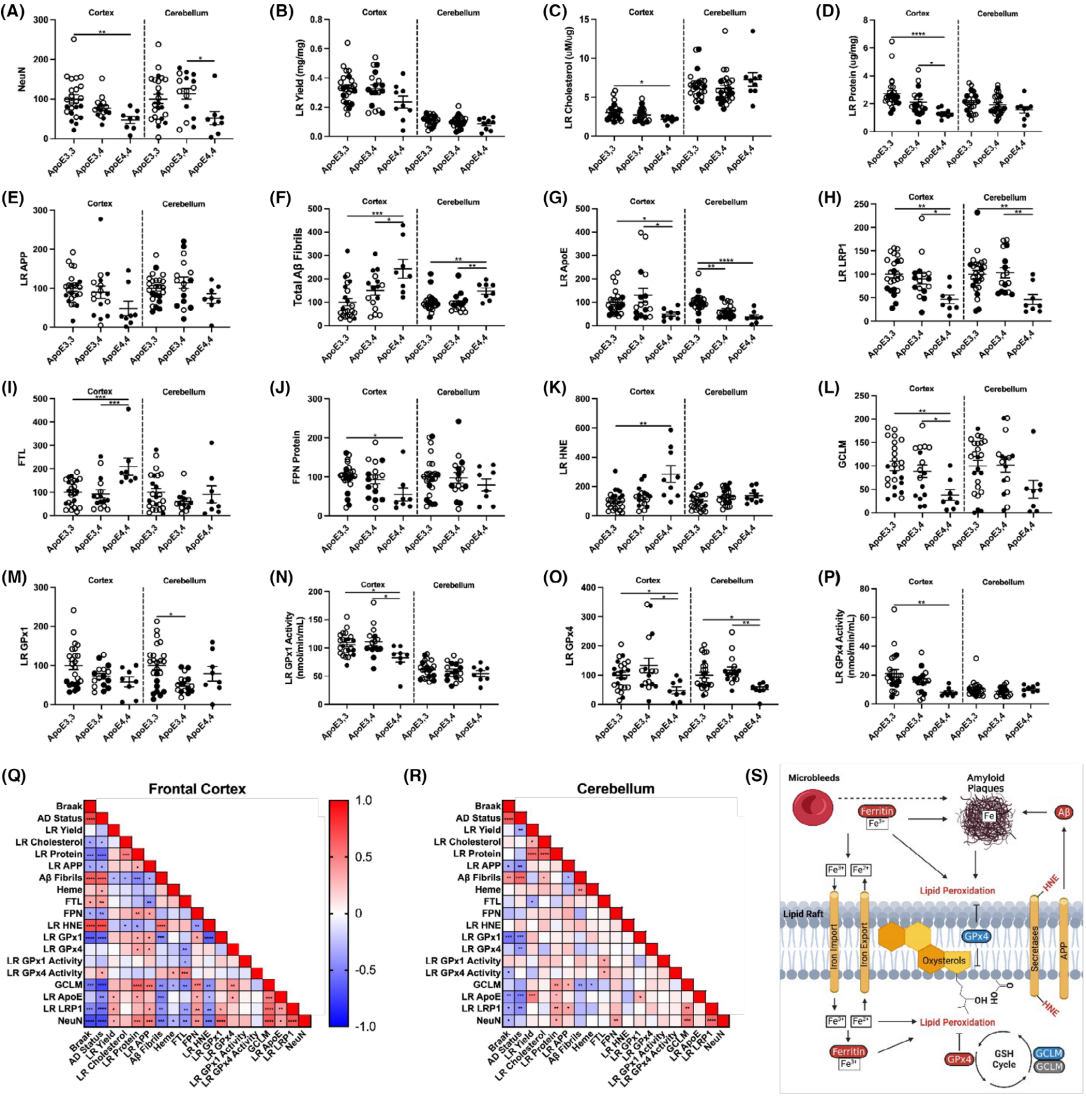
*APOE* allele differences in lipid rafts, antioxidant defense, and amyloid. Levels of (A) NeuN, (B) LR yield, (C) LR cholesterol, (D) total LR protein, (E) LR APP, (F) total Aβ fibrils, (G) LR *APOE*, (H) LR LRP1, (I) FTL, (J) FPN, (K) LR HNE, (L) GCLM, (M) LR GPx1, (N) LR GPx1 activity, (O) LR GPx4, and (P) LR GPx4 activity in prefrontal cortex and cerebellum of cognitively normal (open circles) and demented (closed circles). Correlation matrix of proteins analyzed by *APOE* allele in (Q) frontal cortex and (R) cerebellum. S, Schematic hypothesis linking oxidized lipid rafts to amyloid processing. One‐way analysis of variance with Tukey post hoc, or by Spearman correlation: ^*^ *p* < 0.05, ^**^ *p* < 0.01, ^***^ *p* < 0.001, ^****^ *p* < 0.0001. *APOE*, apolipoprotein E; APP, amyloid precursor protein; FPN, ferroportin; FTL, ferritin light chain; GCLM, glutamate‐cysteine ligase modifier subunit; GPx, glutathione peroxidase; HNE, 4‐hydroxy‐nonenal; LR, lipid raft; LRP1, low‐density lipoprotein receptor‐related protein 1.

Because *APOE* ε4 is associated with greater AD neuropathology, we investigated whether the changes observed in iron metabolism, antioxidant defense to LRs, amyloid processing, and clearance during AD were associated with *APOE* status. Importantly, *APOE* ε4*/*ε4 (ε4*/*ε4) carriers had a significant reduction in NeuN in the cortex, suggesting greater neuronal death is correlated with ε4*/*ε4 (Figure 8A). Interestingly, cerebellum NeuN was also decreased in ε4*/*ε4 carriers despite its reported resistance to AD (Extended Data Figure E8A). In addition, we observed a significant decrease in LR cholesterol and proteins in the ε4*/*ε4 cortex (Figure 8B‐D), suggesting that the composition of LRs is compromised in ε4*/*ε4 carriers. As expected, Aβ fibrils were increased in the ε4*/*ε4 cortex, although total LR APP was unaffected (Figure 8E,F). The cerebellum did not display major changes in LR composition across *APOE* genotypes (Extended Data Figure 8B‐E), although Aβ fibrils were still significantly increased in ε4*/*ε4, again highlighting that although many markers of pathology are not seen in the cerebellum compared to the cortex, it is not completely devoid of all pathological phenotypes.

Next, to determine the consequence of changes in the LR in ε4*/*ε4 carriers, we measured amyloid clearance proteins apoE and LRP1 and saw a significant reduction in *APOE* ε4/ε4 cortex and cerebellum (Figure 8G,H, Extended Data Figure 8G,H). Interestingly, the iron storage protein FTL was highest in the ε4*/*ε4 cortex with corresponding decreases in the iron export protein FPN (Figure 8I,J). Importantly, these changes were directly paralleled with lipid peroxidation in the rafts, as HNE adducts increased significantly in ε4*/*ε4 carriers (Figure 8K). These changes in lipid peroxidation were also observed with corresponding loss of antioxidant capacity, as GCLM, GPx1 activity, and GPx4 levels and activity, although not GPx1 levels, were significantly reduced in ε4*/*ε4 carriers (Figure 8L‐P). Although some of these phenotypes were shared across brain regions, most changes were not seen in the cerebellum (Extended Data Figure 8I‐P). Overall, our data provide strong evidence that many phenotypes associated with iron‐related oxidative damage of lipids, which drive AD pathology, are worse in the ε4*/*ε4 carriers, consistent with the consensus that ε4*/*ε4 carriers are at the highest risk for AD. LR markers correlated negatively with Aβ fibrils in the prefrontal cortex more strongly (Figure 8Q) than in the cerebellum (Extended Data Figure 8R). Aβ fibrils also correlated positively with LR HNE levels. The correlation between NeuN and GCLM or FPN remained strong by *APOE* allele, as well as for clinical “CTL versus AD” and “Braak” stage. LR GPx1 also positively correlated with NeuN.

## 4 DISCUSSION

Iron is increasingly implicated in brain lipid peroxidation during AD.^9^, ^65^, ^66^ This study provides the most comprehensive assessment to date of iron metabolism and oxidative damage in AD brains, highlighting the intricate interplay among iron, antioxidant defenses, lipid peroxidation, and amyloid plaque pathology. Iron accumulation and its redox activity have long been implicated in neurodegenerative diseases, but here, we advance the field by linking regional variations in oxidative damage to specific iron‐related proteins and pathways. This is established by introducing an approach to minimize confounds from vascular entrapped blood by gentle washing that we validated in mouse brains. Examination of the LR and the NRM highlighted that lipid peroxidation predominantly occurs in rafts, which display decreased antioxidants with lower activities. These observations are exacerbated by the *APOE* ε4 allele. The hypothesized role of iron on amyloid pathology was tested in a mouse model using the iron chelator DFO. In ApoEFAD mice, 1 week of DFO treatment solubilized fibrillar amyloid, confirming the role of iron in amyloid plaques. This release of entrapped iron on amyloid fibrils is relevant to ongoing clinical trials in which monoclonal therapies dissolve amyloid plaques but must liberate iron concomitantly.

Heme levels were elevated in AD brains after exogenous blood was minimized. The residual heme may come from leakage from the BBB or microbleeds.^67^, ^68^ Heme iron is liberated by heme oxygenase and stored after the conversion of ferrous iron to ferric by ferroxidase in FTH1^69^ and then packaged on FTL. Because FTL increased with AD, it is indicative of excess iron in AD despite not observing differences in tissue iron. The increases in cellular iron may be harder to discriminate by ICP‐MS and may require more accurate measures capable of subcellular analysis.^70^ Plaques and NFTs also contain ferritin protein and iron,^65^, ^71^, ^72^, ^73^ and iron also directly influences amyloid peptide production by the iron‐response element in the APP primary transcript.^74^ A reexamination of data from Good et al. shows neighboring CA1 neurons without NFTs had 50% less cytoplasmic iron (Table S3 in supporting information).^3^ Additionally, FPN, which is the sole iron export protein, was decreased in both brain regions with AD. Lowered FPN exacerbates ferroptosis in neuroblastoma cells treated with erastin.^75^ Altogether, these data are consistent with the ferroptotic hypothesis that excess iron is a mechanism involved in AD pathogenesis.

In addition to changes in iron and iron‐related pathways, a loss of detoxification pathways and an increase in oxidative damage contribute to AD pathology. Lipid peroxidation can cause the production of reactive a,β‐unsaturated aldehydes with HNE being the most common. Free HNE is cleared through GST family members, primarily GSTA4 in the brain, or ALDH2, which is GSH independent. GSH is needed for most of these processes, which has been reported depleted during AD,^10^, ^76^ likely from the low levels of GCLM shown in these data. GCLM increases the rate of catalysis by 4‐fold for the production of γ‐glutamylcysteine and is the rate‐limiting step in GSH biosynthesis.^59^ Cysteine availability is also rate‐limiting from its primary source, cystine, which is imported by xCT. Independent of GSH, lipid peroxidation is also attenuated by ferroptosis suppressor protein 1 (FSP1) via the quinol cycle,^77^ which we showed is decreased in AD prefrontal cortex and cerebellum. GSH and quinol cycles both rely on NAPDH produced from the pentose phosphate pathway from G6PD and PGD. G6PD is rate‐limiting in the formation of NAPDH^78^ and was also decreased in AD prefrontal cortex. All of these antioxidants and iron metabolic proteins are regulated transcriptionally by Nrf2, which is decreased during AD,^17^, ^79^ also shown here. Decreases in GCLM, FSP1, and G6PD with AD hinder the antioxidant systems that neutralize lipid peroxidation.

We also provide the first analysis of antioxidants in the LRs during AD. LRs are tightly packed microdomains rich in cholesterol, sphingomyelin, and phosphatidylcholine within cellular membranes.^80^, ^81^ Lipid classes within the raft are easily oxidized enzymatically (lipoxygenases) or non‐enzymatically, by Fenton chemistry.^82^ The altered composition of lipid classes within LRs impairs biochemical signal transduction,^83^ which can cause cell death.^84^ Additionally, sterols are more oxidizable than polyunsaturated fatty acids, contrary to prior reports.^85^ Oxidation of lipids within rafts may be responsible for the decreased raft yield we previously identified in AD.^27^ *APOE* ε4 carriers also possess the lowest raft cholesterol, indicative of disrupted rafts potentially inducing cell death.^86^ This introduces a potential mechanism by which the ε4 allele could confer increased AD risk through increased lipid peroxidation and subsequent ferroptosis.

Membrane lipids are protected by GPx4 and Prdx6, which chemically reduce oxidized phospholipids and sterols with GSH.^87^, ^88^ We characterized LRs for glutathione peroxidases (GPx1‐8), which were found to contain only GPx1 and GPx4, but not Prdx6. Prdx6 and GPx1 also reduce lipid hydroperoxides^88^ released from membrane lipids^89^ and may therefore play a secondary role in defense against ferroptosis. The absence of Prdx6 in the LR fraction is consistent with its transient interactions with membranes.^90^, ^91^ AD rafts had lower GPx4 and GPx1 protein and correspondingly lower enzyme activity, consistent with the prominent 2 peroxidation observed in AD. The NRM fraction showed minimal oxidation with AD. Furthermore, baseline GPx4 is two‐fold higher in LRs of normal brains than in the NRM fraction, suggesting that LRs are hotspots for iron‐mediated oxidative damage. The observed increases in whole cell GPx4, but with decreased LR GPx4, suggest a localization issue. Cytosolic GPx4 is 20 kDa, while the 23 kDa mitochondrial isoform contains a leader sequence cleaved for mitochondrial membrane localization.^92^ It is possible leader sequences may be needed for membrane localization. Localization may also be mediated by a phosphorylation site that localizes GPx4 to membranes.^93^

Because LRs are the main site of amyloid production, their lipid peroxidation may alter APP processing enzymes. APP processing is enhanced by the γ‐secretase in rat cortex neurons and human neuronal cells exposed to 1 to 10 µM HNE or 100 µM FeSO_4_.^94^ Moreover, H_2_O_2_ increased BACE1 activity, the primary secretase responsible for amyloid peptide production in mouse neuronal N2a cells transgenic for human APP_695_.^95^ HNE adducts can impact enzyme activities including GCLM, Prdx6, and G6PD, among others relevant to antioxidant defense.^96^ APP processing enzymes contain at least 10% of the amino acids His, Lys, Arg, and Tyr that can be covalently linked by HNE or nitrotyrosine: BACE1 (57/501), PSEN1 (50/467), ADAM10 (142/748). BACE1 activity also increases with AD in the prefrontal cortex^97^ and serum^98^, ^99^ paralleling the increases of lipid peroxidation in serum and tissue.^100^, ^101^ Future studies will evaluate how LR oxidation alters APP processing for amyloid peptides.

AD also decreased Nrf2, a transcriptional regulator of gene responses for oxidative repair that includes glutathione peroxidases and GSH biosynthesis proteins.^102^, ^103^ Nrf2 mRNA was recently shown to be one of the strongest responding genes to ferroptosis.^19^ Levels of Nrf2 mRNA may serve as a marker for ferroptosis in the *post mortem* brain. Furthermore, our data show that Nrf2 levels decline by 75% in the AD cortex but not the cerebellum. Previous work from our coauthors shows a decline of Nrf2‐mediated antioxidant defenses in aging mouse cerebellum.^104^, ^105^ We suspect that the age‐related decline of Nrf2 defense enhances membrane oxidation, which may increase amyloid peptide production from microbleed iron. There were few changes in iron‐related pathways, despite decreased expression of Nrf2‐related genes. However, CD163, the hemoglobin–haptoglobin scavenger that increases in response to microbleeds, was shared between brain regions with AD and was among the top responding mRNAs.^64^ CD163 is primarily found in microglia,^106^ which display high levels of ferritin in AD that may arise from microbleeds.^73^, ^107^, ^108^ Microglia are suggested to initiate the ferroptosis cascade in Parkinson's disease,^14^ which suggests that the present changes in iron signaling and antioxidant defense during AD may extend to other forms of neurodegeneration. Endogenous cell ferritin merits consideration because microglia and astrocytes in young rat brains have 2‐fold more ferritin than neurons, while oligodendroglia have the highest ferritin levels.^70^

New AD changes are shown for multiple transcription factors. While Nrf2 is greatly lowered in AD (see above), there are major increases in NF‐kB and NCOA4. NF‐kB regulates several pro‐oxidant enzymes while NCOA4 responds to iron excess by lysosomal degradation iron‐bound proteins (ferritinophagy).^63^ A relationship of NF‐kB to lipid peroxidation is suggested by its role in ferroptosis of glioblastoma cells treated with a GPx4 inhibitor^109^ and APP/PS1 mice treated with a polyphenol.^110^ Future studies will supersede this bulk analysis, which cannot resolve cell type specificity for iron‐mediated damage.

Finally, *APOE* ε4/ε4 carriers had the greatest oxidative damage in AD brains and lowest antioxidant protection in LRs, which is in contrast to an in vitro study of rat neurons with human *APOE* alleles.^111^ *APOE* alleles may also differentially alter antioxidant gene regulation in the brain consistent with their roles in Aβ clearance and inflammation.^112^ Our findings introduce the *APOE* alleles as important to iron‐mediated processes for further discussion of ferroptosis.

Last, we experimentally tested the role of iron by acute DFO treatment in EFAD mice. Chelation therapy for AD was first attempted in 1991 to remove aluminum by DFO, which slowed cognitive decline for 24 months.^113^ While the cognitive benefits of DFO were attributed to aluminum chelation, Gleason and Bush^114^ also noted that DFO has a 6‐fold greater affinity for ferric iron than aluminum. Our DFO data directly support the major role of iron in multiple AD pathologies, as DFO reduced HNE oxidation and decreased amyloid fibrils. This suggests that iron chelation may solubilize amyloid fibrils to monomers while liberating and chelating iron. Traumatic brain injury, which often results in microbleeds, shows increases in amyloid levels that are attenuated with DFO treatment.^115^ We previously showed that microbleeds precede amyloid formation and that these two pathologies colocalize in the EFAD mice.^116^ Is it possible that amyloid production has a role in preventing more heme‐derived iron from entering and depositing in the brain parenchyma? Furthermore, if iron is liberated from amyloid fibrils, then oxidative damage would be expected in the absence of chelation. DFO treatment increased Nrf2 and downstream antioxidants together with decreased oxidative damage, which support DFO's clinical utility. Current amyloid monoclonal therapies that target aggregated amyloid must also liberate labile iron. The liberated iron could contribute to amyloid‐related imaging abnormalities (ARIAs) in clinical trials.^117^, ^118^, ^119^ Combination therapies should be considered for iron chelators with amyloid monoclonal treatment.

In summary, this analysis of iron metabolism and membrane oxidation in AD is the first to comprehensively document the network of proteins that mediate iron transport and GSH‐mediated neuroprotection relevant to ferroptosis. AD pathway specificity was demonstrated by greater changes observed in the prefrontal cortex than in the cerebellum, consistent with quantitative differences in their AD neuropathology. Our data identify novel alterations in iron‐related transport, clearance, and oxidative repair mechanisms concerning LRs and APP processing. Moreover, multiple anti‐ferroptotic pathways are implicated by the decreases in GCLM, FSP1, FPN, and LR GPx4. Experimental reduction of lipid peroxidation by iron chelation may be considered for co‐therapy with monoclonal antibody treatments to reduce amyloid that inevitably releases entrapped iron associated with amyloid plaques. Taken together, this suite of findings confirms that AD brains contain numerous markers of ferroptosis paralleling the levels of AD neuropathology by brain region and that *APOE* allele and sex modulate the severity of these changes.

## CONFLICT OF INTEREST STATEMENT

The authors declare no conflict of interest. Author disclosures are available in the supporting information.

The authors declare that the data supporting the findings of this study are available within the article and its supplementary information files. Should any raw data files be needed in another format, they are available from the corresponding author upon reasonable request.

## ETHICS STATEMENT

The authors declare no competing interests.

## EXTENDED DATA FIGURE

 [Fig alz14541-fig-0009], [Fig alz14541-fig-0010], [Fig alz14541-fig-0011], [Fig alz14541-fig-0012], [Fig alz14541-fig-0013], [Fig alz14541-fig-0014], [Fig alz14541-fig-0015], [Fig alz14541-fig-0016]

## Supporting information



Supporting Information

Supporting Information
