# Supplementary material for: Iron‐associated lipid peroxidation in Alzheimer's disease is increased in lipid rafts with decreased ferroptosis suppressors, tested by chelation in mice
Source: Alzheimers Dement. 2025 Jan 29;21(1):e14541. doi: 10.1002/alz.14541 (PMC11775463; doi:10.1002/alz.14541)
Supplement: Supplementary file 1 — Supporting Information [file ALZ-21-e14541-s002.docx]

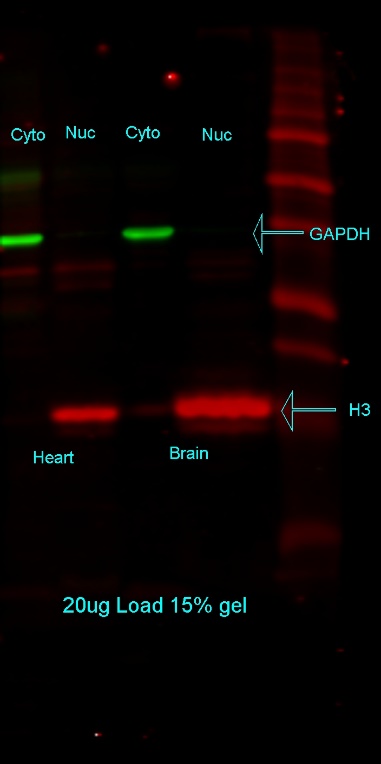


**Supplemental Figure 1:** Nuclear and cytosolic fractions from mouse heart and cortex. Lysates probed by Western blot for histone 3 (H3, nuclear marker) and glyceraldehyde 3-phosphate dehydrogenase (GAPDH, cytosolic marker).


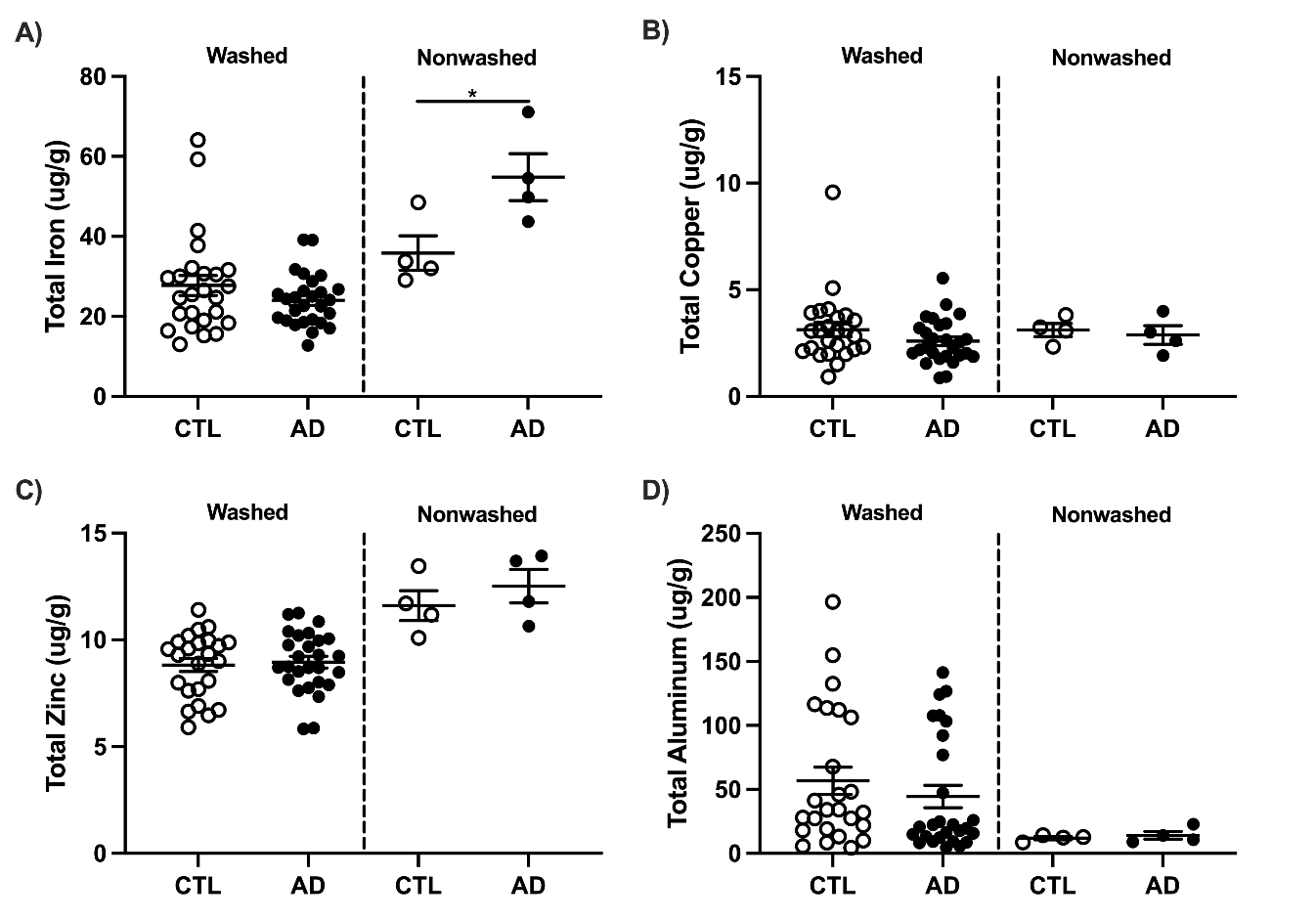


**Supplemental Figure 2:** Total **A)** Iron, **B)** Copper, **C)** Zinc, and **D)** Aluminum concentrations by ICP-MS (Table 1). **A-D,** Human prefrontal cortex was washed with PBS or non-washed. Significance by 2-tailed t-test (CTL vs AD): *p<0.05.

**
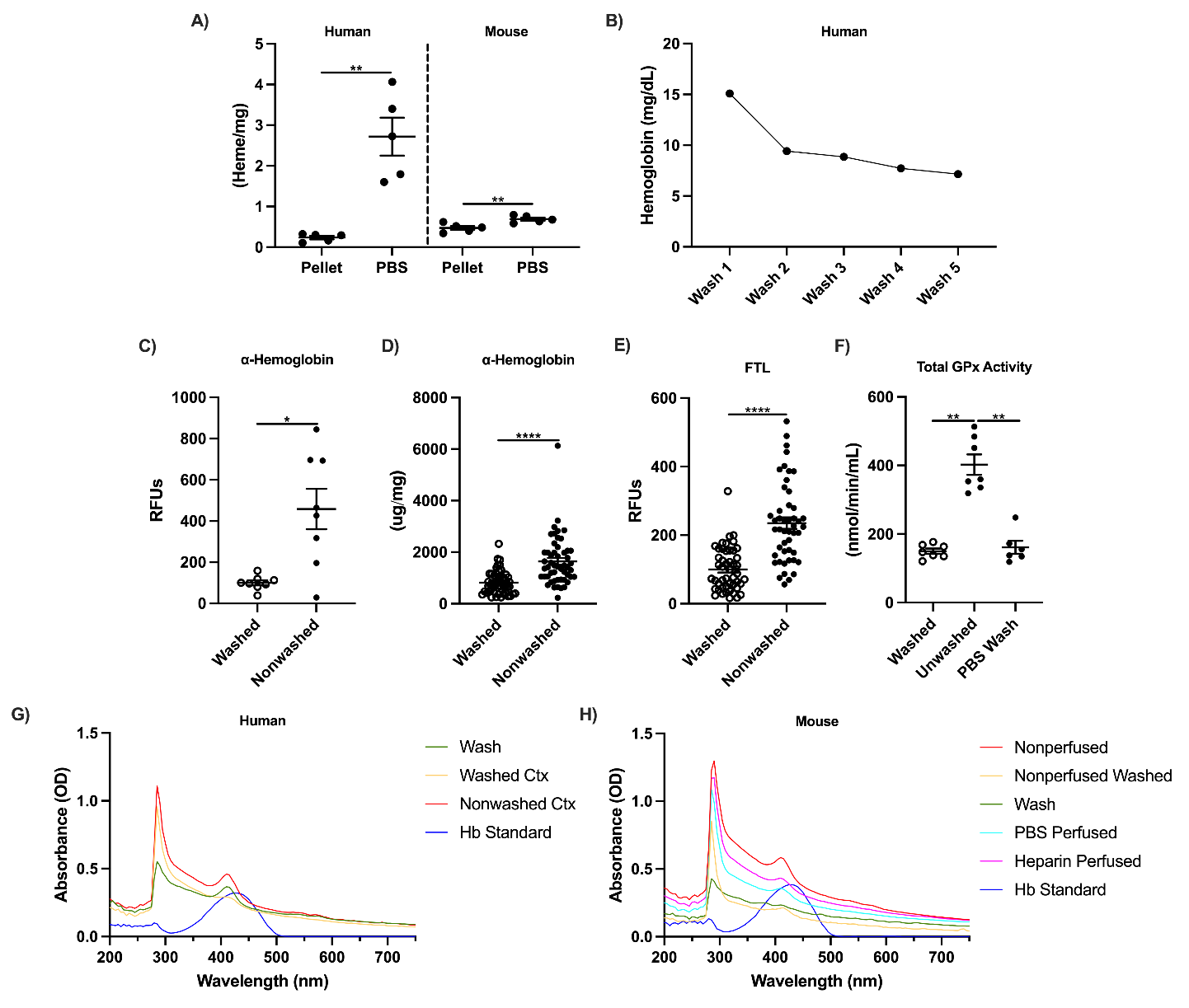
**

**Supplemental Figure 3:** Blood on tissues influences iron and antioxidant measurements. **A)** Nonperfused mouse and washed human cerebral cortex. **B)** Collected wash was assayed for heme in 5 consecutive washes; washes 1 and 2 reduced makers by 90%; washes 3-5 yielded <10% further decreases. The lower α-hemoglobin in washed human frontal cortex was confirmed by **C)** Western blot, **D)** immunoassay, **E)** Western blot for FTL, and **F)** total GPx activity. Significance, 2-tailed t-test (A, C-E) or one-way ANOVA with Tukey’s post-hoc (F): *p<0.05, **p<0.01, ***p<0.001, ****p<0.0001.

**
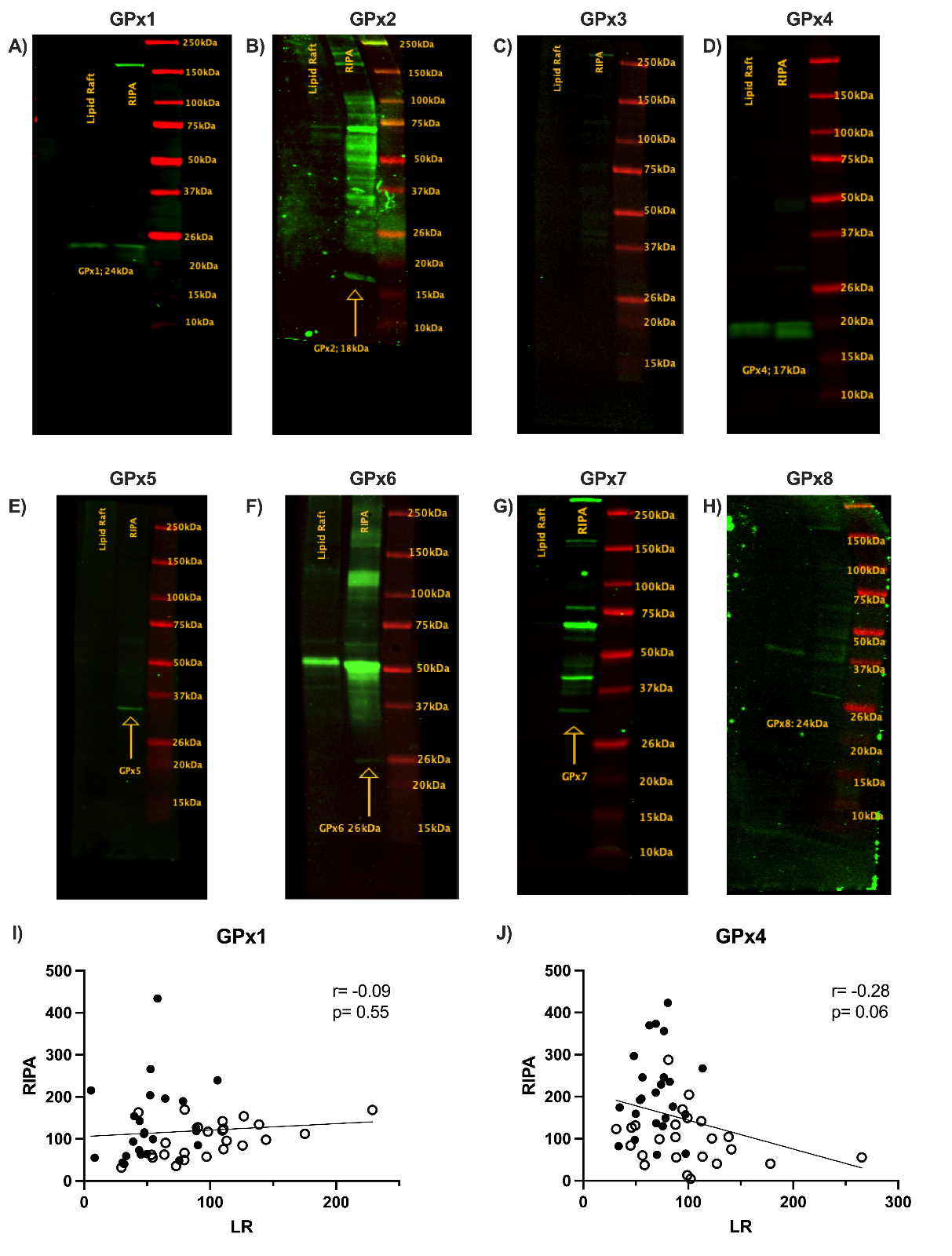
**

**Supplemental Figure 4:** Glutathione peroxidases in lipid rafts from human prefrontal cortex. The GPx isoforms were resolved immunologically on Western blots of lipid raft and whole cell lysates (RIPA buffer extract). **A)** GPx1, **B)** GPx2, **C)** GPx3, **D)** GPx4, **E)** GPx5, **F)** GPx6, **G)** GPx7, **H)** GPx8. Coplots of **I)** GPx1 and **J)** GPx4 for RIPA extract and lipid raft fractions.


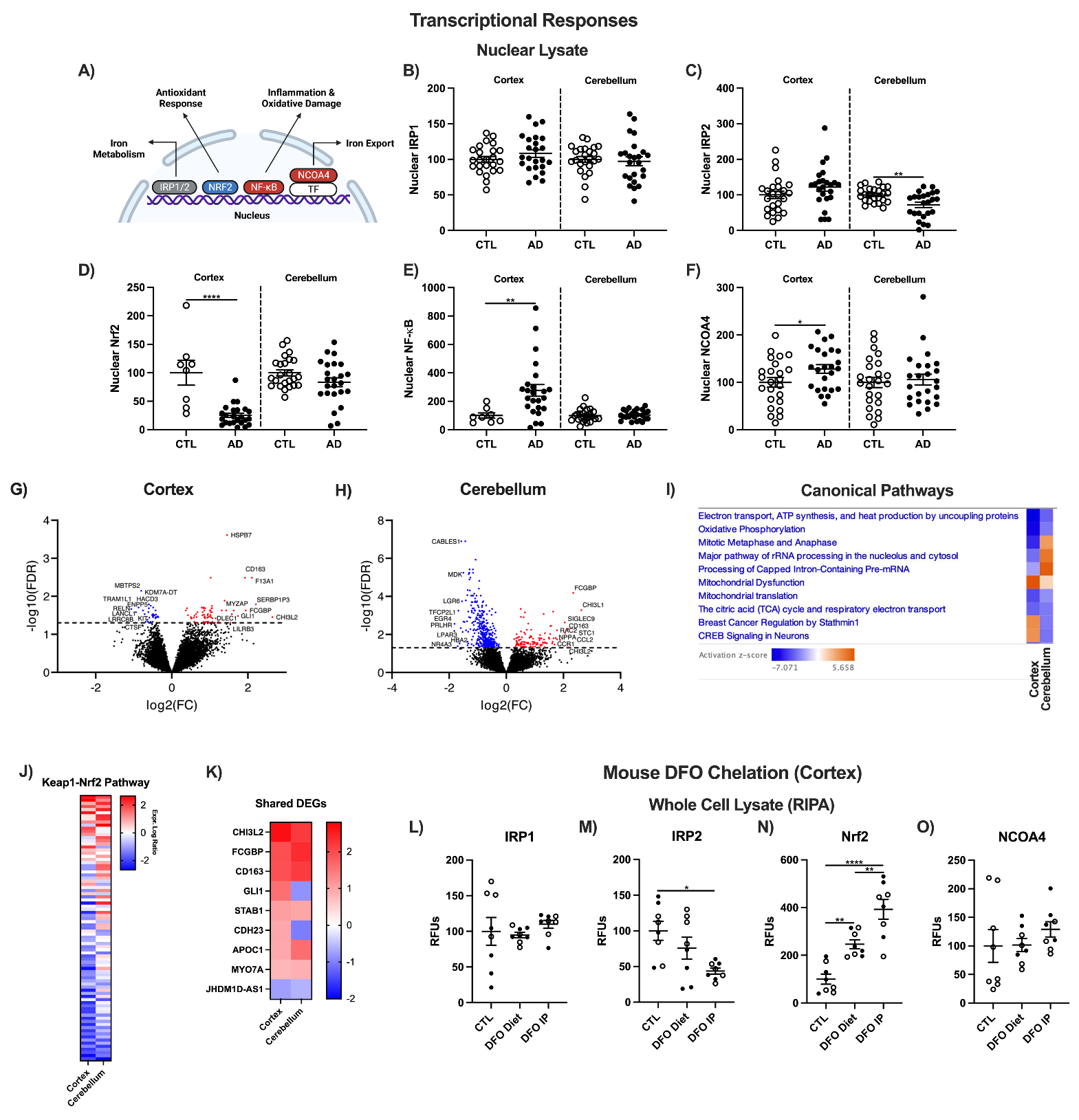


**Supplemental Figure 5:** Transcriptional responses mediating iron homeostasis. **A)** Transcription factors of genes for antioxidant defense and iron signaling in human prefrontal cortex of AD and CTL. IRP, iron regulatory protein; Nrf2, nuclear factor erythroid 2–related factor 2; NF-κB p65, nuclear factor κ-light-chain-enhancer of activated B cells; NCOA4, nuclear receptor coactivator 4; TF, transcription factor. Western blot data from nuclear lysates as relative fluorescent units (RFUs) for **B)** IRP1, **C)** IRP2, **D)** Nrf2, **E)** NF-κB p65, and **F)** NCOA4 in prefrontal cortex and cerebellum. Volcano plots of DEGs from RNA-seq for **G)** prefrontal cortex, **H)** cerebellum, and **I)** top shared canonical pathways. **J)** Keap1- Nrf2 pathway and **K)** shared DEGs between cortex and cerebellum. DFO treated EFAD mouse cortex total (RIPA lysate) for **L)** IRP1, **M)** IRP2, **N)** Nrf2, and **O)** NCOA4 protein in. Significance by 2-tailed t-test (B-F) or One way ANOVA with Tukey’s posthoc test (L-O): *p<0.05, **p<0.01, ****p<0.0001.

| **Age** | **Sex** | **AD Status** | **Braak** | **ApoE Allele** | **PMI (hours)** | **Frontal Cortex** | **Cerebellum** | **Race** | **MMSE** | **MMSE Interval (Months)** | **ADRC** |
| --- | --- | --- | --- | --- | --- | --- | --- | --- | --- | --- | --- |
| 99 | Female | CTL | 0 | 3,3 | 9 | x | x | White | 28 | 23.4 | USC |
| 91 | Female | CTL | 0 | 3,3 | 8.75 | x | x | White | 20 | 84 | USC |
| 85 | Female | CTL | 0 | 3,3 | 7 | x | x | White | N/A | N/A | USC |
| 95 | Female | CTL | 1 | 3,3 | 3.25 | x | x | White | N/A | N/A | USC |
| 95 | Female | CTL | 2 | 3,3 | 2.92 | x | x | Hispanic | N/A | N/A | UCI |
| 87 | Female | CTL | 2 | 3,3 | 10.75 | x | x | White | 29 | 1 | USC |
| 86 | Female | CTL | 3 | 3,3 | 6.17 | x | x | White | 30 | 35.5 | UCI |
| 90 | Female | CTL | 3 | 3,3 | 2.2 | x | x | White | N/A | N/A | UCI |
| 89 | Female | CTL | 3 | 3,3 | 3.58 | x | N/A | White | 24 | 5.9 | UCI |
| 94 | Female | AD | 4 | 3,3 | 10.5 | x | x | Black | N/A | N/A | USC |
| 81 | Female | AD | 5 | 3,3 | 7.5 | x | x | Hispanic | N/A | N/A | USC |
| 89 | Female | AD | 5 | 3,3 | 1.5 | x | x | White | N/A | N/A | USC |
| 66 | Female | AD | 6 | 3,3 | 17 | x | x | White | N/A | N/A | USC |
| 70 | Female | CTL | 1 | 3,4 | 4.17 | N/A | x | White | 28 | 20.1 | UW |
| 91 | Female | CTL | 3 | 3,4 | 3.33 | x | x | White | 27 | 42.5 | UCI |
| 87 | Female | CTL | 3 | 3,4 | 4.57 | x | x | White | 29 | 85.6 | UCI |
| 97 | Female | CTL | 3 | 3,4 | 0 | N/A | x | N/A | 26 | 6.6 | UW |
| 91 | Female | CTL | 3 | 3,4 | 11.2 | x | x | N/A | 23 | 10.1 | UW |
| 86 | Female | CTL | 3 | 3,4 | 9.33 | N/A | x | White | 29 | 12.3 | UW |
| 85 | Female | CTL | 3 | 3,4 | 4.2 | N/A | x | White | 29 | 37.9 | UW |
| 89 | Female | CTL | 3 | 3,4 | 3.5 | N/A | x | White | 27 | 28.7 | UW |
| 74 | Female | CTL | 3 | 3,4 | 4.5 | N/A | x | White | 30 | 44.3 | UW |
| 93 | Female | CTL | 5 | 3,4 | 4.15 | x | x | White | 30 | 81.6 | UCI |
| 81 | Female | AD | 5 | 3,4 | 5.5 | x | x | White | N/A | N/A | USC |
| 84 | Female | AD | 5 | 3,4 | 6 | x | x | White | N/A | N/A | USC |
| 80 | Female | AD | 5 | 3,4 | 19 | x | x | White | N/A | N/A | USC |
| 96 | Female | AD | 6 | 3,4 | 9 | N/A | x | Black | N/A | N/A | USC |
| 87 | Female | AD | 6 | 3,4 | 4.5 | N/A | x | N/A | N/A | N/A | USC |
| 77 | Female | AD | 6 | 3,4 | 8.25 | x | x | White | N/A | N/A | USC |
| 78 | Female | AD | 4 | 4,4 | 15.5 | x | x | White | N/A | N/A | USC |
| 81 | Female | AD | 5 | 4,4 | 4 | x | x | White | N/A | N/A | USC |
| 80 | Female | AD | 5 | 4,4 | 3.75 | x | x | White | N/A | N/A | USC |
| 76 | Female | AD | 5 | 4,4 | 5.25 | x | x | White | N/A | N/A | USC |
| 76 | Male | CTL | 0 | 3,3 | 11.25 | x | x | Hispanic | N/A | N/A | USC |
| 82 | Male | CTL | 0 | 3,3 | 9 | x | x | White | 28 | N/A | USC |
| 93 | Male | CTL | 0 | 3,3 | 12 | x | x | White | 30 | 1229.2 | USC |
| 93 | Male | CTL | 0 | 3,3 | 3.75 | x | x | White | N/A | N/A | USC |
| 86 | Male | CTL | 2 | 3,3 | 4.42 | x | x | White | 25 | 48.4 | UCI |
| 83 | Male | CTL | 2 | 3,3 | 3.18 | x | x | White | N/A | N/A | UCI |
| 86 | Male | CTL | 3 | 3,3 | 2.92 | x | x | White | 20 | 37 | UCI |
| 80 | Male | CTL | 3 | 3,3 | 4.05 | x | x | White | 26 | 95.1 | UCI |
| 97 | Male | AD | 3 | 3,3 | 5.25 | x | x | Hispanic | N/A | N/A | USC |
| 87 | Male | AD | 4 | 3,3 | 4.75 | x | x | White | N/A | N/A | USC |
| 76 | Male | AD | 5 | 3,3 | 9.75 | x | x | Hispanic | N/A | N/A | USC |
| 88 | Male | AD | 5 | 3,3 | 6.75 | x | x | Hispanic | N/A | N/A | USC |
| 92 | Male | CTL | 0 | 3,4 | 5.25 | x | x | White | N/A | N/A | USC |
| 87 | Male | CTL | 1 | 3,4 | 4 | N/A | x | White | 25 | 8.9 | UW |
| 77 | Male | AD | 2 | 3,4 | 6 | x | x | White | N/A | N/A | USC |
| 90 | Male | CTL | 3 | 3,4 | 5.8 | x | x | White | N/A | N/A | UCI |
| 81 | Male | AD | 3 | 3,4 | 7.25 | x | x | Hispanic | N/A | N/A | USC |
| 97 | Male | CTL | 4 | 3,4 | 5.83 | x | x | White | 26 | 7 | UCI |
| 87 | Male | CTL | 4 | 3,4 | 6.28 | x | x | White | 22 | 101.5 | UCI |
| 85 | Male | AD | 5 | 3,4 | 7.25 | x | x | Hispanic | N/A | N/A | USC |
| 72 | Male | AD | 6 | 3,4 | 9 | x | x | White | N/A | N/A | USC |
| 75 | Male | AD | 0 | 4,4 | 9.5 | x | x | White | N/A | N/A | USC |
| 81 | Male | AD | 5 | 4,4 | 6.54 | N/A | x | Asian | N/A | N/A | USC |
| 88 | Male | AD | 5 | 4,4 | 5 | x | x | White | N/A | N/A | USC |
| 92 | Male | AD | 5 | 4,4 | 8.25 | x | x | White | N/A | N/A | USC |
| 70 | Male | AD | 5 | 4,4 | 3.5 | x | x | White | N/A | N/A | USC |

**Supplemental Table 1:** Data shown for brain specimens by each ADRC source: USC, 37; UCI, 14; UW, 8. White, 76%; Latino, 14%; African American, 3%; Asian, 2%; N/A, 5%. ApoE3,3, 42%, ApoE3,4, 42%, ApoE4,4, 16%.

| **Measurement** | **Cortex**  **(AD vs CTL)** | **Cerebellum**  **(AD vs CTL)** | **Cortex**  **(ApoE3,3 vs 3,4 & 4,4)** | **Cerebellum**  **(ApoE3,3 vs 3,4 & 4,4)** | **Cortex**  **Male**  **(AD vs CTL)** | **Cortex**  **Female**  **(AD vs CTL)** |
| --- | --- | --- | --- | --- | --- | --- |
| **FTL** | +65% ** | +10%, n.s. | +30%, n.s. | -30%, n.s. | +55%, n.s. | +75% * |
| **FSP1** | -40% **** | -30% **** | -40% * | -20% ** | -50%, n.s. | -30% * |
| **GCLM** | -60% **** | -35% * | -30%, n.s. | -15%, n.s. | -65% **** | -55% *** |
| **GPx4** | +110% **** | -15% * | +45% * | -30, n.s. | +45%, n.s. | +210% *** |
| **GPx4, LR** | -30% ** | -40% **** | -5%, n.s. | -5%, n.s. | -25% ** | -35%, n.s. |
| **GPx4 Activity, LR** | -40% * | -10%, n.s. | -50% ** | -15%, n.s. | -40%, n.s. | -40%, n.s. |
| **Heme** | +25% * | +30% ** | +5%, n.s. | +10%, n.s. | +20%, n.s. | +25%, n.s. |
| **HNE, Total** | +60% **** | -20%, n.s. | +35% ** | -25% * | +35% ** | +90% *** |
| **HNE, LR** | +160% **** | +45% ** | +80% ** | +35% * | +170% ** | +180% *** |
| **NF-κB, nuclear** | +175% ** | +5%, n.s. | +70%, n.s. | +5%, n.s. | +45%, n.s. | +290% ** |
| **Nrf2, nuclear** | -75% **** | -15%, n.s, | -65% *** | -15%, n.s. | -85% ** | -60% ** |

**Supplemental Table 2:** AD change in prefrontal cortex (Brodmann 8-10) and cerebellum by ApoE3 and 4 alleles. FTL, ferritin light chain; FSP1, ferroptosis suppressor protein 1; GPx4, glutathione peroxidase 4. t-test *p<0.05, **p<0.01, ***p<0.001, ****p<0.0001; n.s, not significant.

|  | **NFT-Bearing Neurons** | | | | **NFT-Free Neurons** | | |
| --- | --- | --- | --- | --- | --- | --- | --- |
|  | NFT | Cytoplasm | Nucleus | Neuropil | Cytoplasm | Nucleus | Neuropil |
| **AD** | 65 ± 9 | 20 ± 3 | 42 ± 5 | 14 ± 3 | 12 ± 2* | 44 ± 7* | 11 ± 1* |
| **CTL** | N/A | N/A | N/A | N/A | 4 ± 0.5 | 17 ± 2 | 4 ± 0.5 |

**Supplemental Table 3:** Iron in NFT free and bearing neurons presented as mass charge ratio (m/z) by Good et al. 1992^45^. Significance analyzed in this report by 2-tailed t-test: *p<0.05.
